# Encapsulation of Pomegranate Peel Phenolic Extract Using Orange Peel and Pectin: Stability Evaluation in Baked Products

**DOI:** 10.1002/fsn3.72311

**Published:** 2026-09-02

**Authors:** Marwa Ezz El‐Din Ibrahim, Randah M. Alqurashi

**Affiliations:** ^1^ Department of Nutrition and Food Science College of Home Economic, Capital University Cairo Egypt; ^2^ Department of Food and Nutrition Sciences, College of Agriculture and Food Sciences King Faisal University Al‐Ahsa Saudi Arabia

**Keywords:** antioxidant, commercial pectin, encapsulation, lipid oxidation, lyophilization, pomegranate extract, thermal stability

## Abstract

This study investigated a patented two‐stage lyophilization technique to encapsulate phenolic extract from pomegranate peel using orange peel and commercial pectin as carrier materials. The aim was to improve the stability of bioactive compounds during storage and thermal processing in baked products. Pomegranate peel extract was encapsulated using two approaches: a two‐stage lyophilization method with orange peel at a 7:1 extract‐to‐wall ratio and a conventional single‐stage freeze‐drying method with pectin. Encapsulated and non‐encapsulated extracts were characterized by HPLC, evaluated for color stability during accelerated storage (60°C for 45 days), and incorporated into crackers (1000 ppm) to examine thermal stability, lipid oxidation, fiber enrichment, and sensory attributes. HPLC analysis identified 17–18 phenolic compounds across the different extracts, with punicalagin as the predominant phenolic compound. Compared with commercial pectin, the orange peel‐based encapsulation system showed superior retention of phenolic compounds during storage. Following baking, phenolic loss was only 6.7% in the orange peel system, compared with 40.8% and 69.4% in the non‐encapsulated and pectin systems, respectively. Encapsulation also improved color stability, reduced lipid oxidation, increased dietary fiber, and maintained sensory acceptability of fortified crackers. Overall, the patented orange peel‐based encapsulation system demonstrated superior protection of pomegranate peel phenolic compounds compared with the conventional pectin‐based system, highlighting the potential of citrus by‐products as sustainable natural carrier materials for developing functional bakery products.

## Graphical abstract

Raw materials: Natural wall materials. Extraction: Pomegranate in ethanol (80%), 1:10 ratio, 2 days, 4°C, Phenolic extract preparation. Encapsulation: Two‐Stage Lyophilization (Orange Peel), Stage 1: Mix extract + orange peel (4:1); −40°C, 24 h, Stage 2: Re‐mix (3:1); −40°C, 24 h, 7:1, Single‐stage lyophilization (Commercial Pectin), Mix extract + pectin (7:1), Heat 3 min (70°C), Freeze‐dry −40°C, 24 h. Application: Fortified Crackers (1000 ppm), Baking Oven (7 min at 200°C). Key Results: Capsules, Pomegranate extract PE, Orange capsules, Pectin, Total Phenolic content: ↑75% (Orange) versus ↑63% (pectin) versus ↑40.5% (PE) after storage, Antioxidant activity: > 90% maintained (Orange system), Crackers, Phenolic retention: Crackers: loss 6.7% (Orange) versus 69.4% (Pectin) versus 40.8% non‐encapsulated after baking, Reduced Lipid Oxidation, Fiber Enrichment.

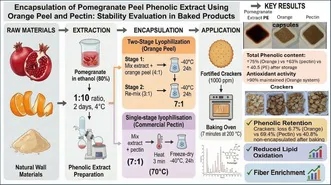

## 1 Introduction

Food processing industries generate large quantities of by‐products that can be utilized as valuable sources of bioactive compounds. Among these by‐products, pomegranate ( *Punica granatum* L.) peel represents approximately 40%–50% of the total fruit weight and is commonly discarded during juice production. According to the Food and Agriculture Organization (FAO) estimates, industrial pomegranate‐processing centers generate approximately 1.5 million tons of waste annually (Huang et al. 2025). Despite being treated as waste, pomegranate peel contains considerable amounts of phenolic compounds such as ellagitannins, gallotannins, flavonoids, and anthocyanins (Singh et al. 2023; Toledo‐Merma et al. 2022). These compounds are widely recognized for their antioxidant, antimicrobial, and anti‐inflammatory activities and therefore have potential for application in functional food systems (Rahul et al. 2025).

The direct incorporation of pomegranate peel extract into food products, however, presents several limitations. Phenolic compounds are sensitive to environmental conditions including oxygen, light, temperature, and pH, which can lead to degradation and a reduction in their biological activity (Cao et al. 2021). In addition, the bitter and astringent taste associated with pomegranate peel extract may negatively affect the sensory acceptability of fortified foods (Kaderides et al. 2021). For these reasons, encapsulation technologies have been widely explored as a strategy to protect phenolic compounds, improve their stability, and reduce undesirable sensory effects (Akonjuen and Aryee 2023; Choudhury et al. 2021; Cortez‐Trejo et al. 2021).

Spray drying remains one of the most commonly used techniques for industrial encapsulation because of its relatively low cost and suitability for large‐scale production (Insang et al. 2022). Several studies have examined the encapsulation of pomegranate extracts using different wall materials, including maltodextrin, gum arabic, whey protein, and citrus‐derived polysaccharides (Kaderides et al. 2020; Sanhueza et al. 2022). Nevertheless, the high inlet temperatures typically used during spray drying (145°C–160°C) can lead to considerable losses of heat‐sensitive phenolic compounds, sometimes reaching 30%–40% (Stabrauskiene et al. 2024). Rapid moisture removal during the process may also affect the structure of the wall material and influence the protection and release of the encapsulated compounds (Da Silva Júnior et al. 2023).

Freeze‐drying (lyophilization) has therefore been proposed as an alternative encapsulation approach. This technique operates at low temperatures under vacuum, allowing water removal by sublimation and thereby reducing thermal degradation of sensitive compounds while producing porous structures that may facilitate controlled release (Ledari et al. 2024). However, lyophilization also has some limitations, including its relatively high operating cost, long processing time, and high energy consumption, which may restrict its large‐scale industrial application despite its excellent preservation efficiency (Ratti 2001; Tontul and Topuz 2017). The effectiveness of encapsulation systems is strongly influenced by the type of wall material used. Commercial pectin, commonly extracted from citrus peels and apple pomace, is frequently employed due to its film‐forming ability, biocompatibility, and gel‐forming properties (Assadpour et al. 2017; Esfanjani et al. 2015). Several studies have demonstrated the capacity of pectin‐based systems to protect phenolic compounds, although their performance may vary depending on the encapsulation technique (Ghasemi et al. 2017; Robert et al. 2015). Pectin has also been used in different encapsulation approaches, including nano spray drying, nanocomplex formation, and coacervation (Fathi et al. 2014). In addition to conventional carriers, citrus processing by‐products such as orange peel have attracted attention as potential natural encapsulating materials. Orange peel contains dietary fiber, endogenous pectin, and phenolic compounds that may contribute to improved protection of bioactive substances (Liu et al. 2025; Panić et al. 2021).

Most previous studies have employed single‐stage encapsulation processes in which the core material is directly mixed with the wall material before drying (Ballesteros et al. 2017; Bińkowska et al. 2024). Although this approach is widely used, its protective performance depends largely on the properties of the wall material and the encapsulation technique employed, which may influence the stability and release behavior of encapsulated bioactive compounds (Fathi et al. 2014; Bińkowska et al. 2024). To overcome the limitations of this approach, a two‐stage lyophilization method was recently developed and patented by Ibrahim and Alqurashi (2024). In this method, orange peel is incorporated sequentially as a wall material, first forming a primary matrix with the pomegranate peel extract, followed by the formation of a second protective layer. The resulting dual‐layer structure is expected to enhance the resistance of encapsulated phenolic compounds to environmental stressors such as oxygen, light, temperature, and moisture.

While spray‐drying encapsulation of pomegranate extracts has been extensively studied (Da Silva Júnior et al. 2023; Kaderides et al. 2020), limited research has focused on lyophilization‐based encapsulation of pomegranate peel extracts using natural wall materials. More importantly, no previous studies have investigated the effectiveness of the two‐stage lyophilization method. The comparative performance of orange peel under this two‐stage lyophilization versus commercial pectin under conventional single‐stage freeze‐drying has not been evaluated. This comparison allows for assessing the encapsulation efficiency of the patented orange peel system relative to a widely used encapsulation material under standard lyophilization conditions. This study aimed to evaluate a patented two‐stage lyophilization encapsulation method for pomegranate peel phenolic extract using orange peel and commercial pectin as wall materials. In addition, the effectiveness of these encapsulation systems in improving phenolic stability during storage and baking was investigated. This research represents the first comprehensive evaluation of a two‐stage lyophilization encapsulation method for phenolic‐rich extracts using natural wall materials. The findings contribute to the development of novel encapsulation strategies that support sustainable food innovation through the valorization of industry by‐products.

## 2 Materials and Methods

### 2.1 Materials

Pomegranate ( *Punica granatum* L.), which was harvested in September 2023, was kindly provided from the Assiut region in the south of Egypt and exported to the Al‐Ahsa market in Saudi Arabia. In our research, pomegranate was obtained from a local market. Then the pomegranate was peeled in the laboratory. The peels were dried at 40°C for 48 h and kept at −30°C until use. The peels were ground in a laboratory mill (FOSS Cyclotec 1093, China) immediately before extraction. The particle size distribution of the milled pomace was determined by a sieve shaker. The particle mean diameter was about 0.1 mm (Kaderides et al. 2020).

Orange balady ( *Citrus sinensis* ), which was harvested in December 2023, was kindly provided from the Nile Delta in Egypt and exported to the Al‐Ahsa market in Saudi Arabia. In our research, a juice extractor discharged the rind, and the inner part of the fruit was pressed. After the oranges were thoroughly squeezed, the peel was removed, and the outer layer was kept of the white inner fiber part of the peel. Orange waste fiber was ground to a high particle size and soaked in hot water (90°C) for 20 min to produce a fiber with high water holding capacity (Kaderides and Goula 2017). The samples were dehydrated in a tray dryer at 40°C until the weight remained constant and kept at −15°C until use.

The commercial citrus pectin (from *Citrus sinensis* or citrus peel) was purchased from Sigma‐Aldrich (St. Louis, MO, USA). Pectin was stored in a freezer (VALUETEC, China; model VRD499G1) at −15°C until use.

### 2.2 Extraction of Phenolic Extract From Pomegranate

Dried powder of pomegranate peels was soaked in 80% ethanol at a ratio of 1:10 g/ml and kept in a refrigerator with daily shaking for 2 days. This was followed by centrifugation at 10,000 rpm for 20 min. The ethanolic extract was concentrated under reduced pressure using a vacuum rotatory evaporator (Buchi Rotavapor R‐210, Flawil, Switzerland) at 50°C; every 100 mL was evaporated to 20 mL. A part of the evaporated ethanolic extract was kept for capsule preparation; another part was dried by freeze‐drying machine (Martin Christ Gefriertrocknungsanlagen GmbH, Germany); the samples were previously frozen and then put into a chamber at −60°C under pressure of 0.05 bar, being maintained under these conditions for 48 h (Ballesteros et al. 2017). The dry extract was rehydrated with a few drops of distilled water to obtain a semi‐solid crude extract.

### 2.3 Encapsulation of Phenolic Extract in Orange Peel

A patented two‐stage encapsulation method developed by Ibrahim and Alqurashi (2024) was employed in this study. Orange peel powder was used as a natural wall material, and phenolic extract derived from pomegranate peel served as the core material. The encapsulation process consisted of two sequential freeze‐drying stages, with a total extract‐to‐wall material ratio of 7:1. In the **first stage**, the phenolic extract was mixed with dried orange peel powder at a ratio of 4:1 (extract: wall material). The mixture was manually mixed until complete absorption of the extract was achieved, pre‐frozen using a deep freezer (TSX Series, Thermo Scientific, USA) at −40°C for 24 h and then subjected to freeze‐drying at −40°C under a vacuum pressure of 0.05 bar for 24 h. In the **second stage**, the freeze‐dried powder obtained from the first cycle was re‐mixed with additional phenolic extract at a ratio of 3:1 (extract: encapsulated powder), and the freeze‐drying process was repeated under the same conditions. The final encapsulated product was stored in a desiccator until further analysis.

### 2.4 Encapsulation of Phenolic Extract in Pectin

Coating for the encapsulation process was made from pectin as wall material with some modifications as described by (Saenz et al. 2009). The coating was made by dissolving pectin in evaporated pomegranate ethanolic extract prepared above, with a ratio of pomegranate ethanolic extract to pectin of 7:1, then heated at 70°C for 3 min while stirring using a hot plate magnetic (Model NO. MC‐MS‐H380 Pro, Guangzhou). Then, the samples were pre‐frozen using a deep freezer (TSX Series, Thermo Scientific, USA) at −40°C for 24 h and then subjected to freeze‐drying by a pilot‐scale freeze dryer at −40°C. The complete freeze‐drying process was done in 24 h. The encapsulated powder was stocked into a desiccator.

### 2.5 Identification and Quantification of Phenolic Compounds

Phenolic compounds in non‐encapsulated pomegranate extract (PE) and encapsulated extracts (encapsulated pomegranate extract in orange peel EPEOP and encapsulated pomegranate extract in pectin EPEP) were analyzed using high‐performance liquid chromatography (HPLC). For HPLC analysis, samples were finely pulverized before extraction. A 0.5 g portion of each sample was transferred into a 50‐mL centrifuge tube and extracted with 10 mL of 80% methanol according to Alqurashi et al. (2016). The mixture was shaken for 30 min and centrifuged at 10,000×*g* for 10 min. The supernatant was filtered through a 0.45‐μm membrane filter before injection. HPLC analysis was carried out using an Agilent 1260 series system equipped with a Zorbax Eclipse Plus C8 column (4.6 × 250 mm, 5 μm). The mobile phase consisted of water (A) and 0.05% trifluoroacetic acid in acetonitrile (B) at a flow rate of 0.9 mL/min. The gradient program was as follows: 0 min (82% A); 0–1 min (82% A); 1–11 min (75% A); 11–18 min (60% A); 18–22 min (82% A); and 22–24 min (82% A). Detection was performed at 280 nm, with an injection volume of 5 μL and a column temperature of 40°C. Phenolic compounds were identified by comparing their retention times with those of the corresponding phenolic standards. Quantitative determination of the identified phenolic compounds was performed using phenolic standard compounds following the approach described by Khoddami et al. (2013). HPLC quantification was based on external standards; however, full analytical method validation, including calibration curve parameters, limits of detection and quantification (LOD and LOQ), and recovery, was not performed in the present study. The chromatographic conditions were adapted and optimized based on previously reported methods (Bae et al. 2015). The HPLC results were intended to characterize the phenolic profile of each extract; therefore, they are presented descriptively and were not subjected to statistical comparison.

### 2.6 Color Characteristics of Nonencapsulated and Encapsulated Extracts

The color characteristics of the nonencapsulated pomegranate peel extract (PE) and the encapsulated extracts (EPEOP and EPEP) were evaluated both before and after storage at 60°C for 45 days. Measurements were performed in triplicate at each sampling point. Color determination was conducted using a spectral colorimeter (MiniScan, HunterLab, Reston, Virginia, USA) according to the CIE L*a*b* color system (Commission Internationale de l'Éclairage). This system is based on three parameters: L* (lightness), a* (red‐green coordinate), and b* (yellow‐blue coordinate) (Sapers and Douglas 1987).

### 2.7 Storage Stability of Phenolic Extracts

The nonencapsulated PE and encapsulated extracts (EPEOP and EPEP) were evaluated for total phenolic content and antioxidant activity during storage at 60°C for 45 days. The samples were stored in an oven (Memmert, GmbH+Co.KG 8540 Schwabach W‐Germany) for accelerated shelf‐life testing. Triplicate samples were taken every 3 days to determine total phenolic content and antioxidant activity.

The total phenolic content was assessed by applying the Folin–Ciocalteu method (Tsali and Goula 2018) and using gallic acid as a standard (SOMATCO, Saudi Arabia). The antioxidant activity of the samples was determined using a 1,1‐diphenyl‐2‐picrylhydrazine (DPPH) radical scavenging assay (Tsali and Goula 2018). The result was calculated as a percentage using the following equation:

$$\text{DPPH scavenged}\;\left(\%\right)=\left[\left(A_{\mathrm{con}}-A_{\text{test}}\right)/A_{\mathrm{con}}\right]\times 100$$

*A*_con_ is the absorbance of the control reaction (DPPH), and *A*_test_ is the absorbance of the samples.

Total phenolic content and antioxidant activity were determined on the samples as analyzed. The encapsulated extracts were analyzed in their freeze‐dried powder form, whereas the non‐encapsulated pomegranate peel extract was analyzed as a concentrated semi‐solid extract obtained after solvent evaporation.

### 2.8 Fortification of Crackers With Phenolic Compounds

The following ingredients were used to make the crackers dough: 270 g of flour, 70 g of butter, 1 g of salt, and 2 g of NaHCO_3_. Following the addition of the butter and salt, the flour was combined for an additional 0.5 min at moderate speed. The machine was filled with the remaining ingredients, shut, and the dough was stirred for 15 min. The dough sample was mixed and then left to rest in covered containers for 30 min. Samples of crackers were fortified with nonencapsulated PE and encapsulated extract (EPEOP and EPEP) at a phenolic concentration of 1000 ppm (w/w) for flour weight (pomegranate extract 22.16 g; encapsulated extract from orange peel capsule, 20.34 g; encapsulated extract from pectin capsule, 19.60 g). The prepared cracker dough was baked in an oven for 7 min at 200°C, cooled for 30 min, and then packaged in high‐density polyethylene.

To evaluate the effect of storage duration on phenolic stability, antioxidant activity, lipid oxidative deterioration, and color changes, control and enriched crackers were stored in polyethylene bags at 60°C for 24 days. Every 3 days, three independent samples (3 g each) were collected from each treatment for analysis. The color of the upper surface of the crackers was measured in triplicate using the method described above. Polyphenols were extracted from cracker samples using the ultrasonic‐assisted extraction method according to (Kaderides et al. 2015). Total phenolic content and antioxidant activity of the cracker extracts were determined following the procedures described in Section 2.7, and all analytical measurements were performed in triplicate. The cracker samples were analyzed in their baked form as prepared, and the results were reported on an as‐analyzed basis. To assess the protective performance of the encapsulated extracts within the food matrix during thermal processing, salted cracker samples were analyzed before and after baking for phenolic content and antioxidant activity.

### 2.9 Peroxide Value (PV)

Crackers were defatted twice with 15 mL of hexane and evaporated on an electric Hot Plate Machine (Model NO. MC‐MS‐H380 Pro, Guangzhou) for the oxidation analysis. In accordance with the procedure outlined in (Kaderides et al. 2015), hexane was evaporated, and the recovered oil was utilized to assess the peroxide value PV. After adding Na_2_S_2_O3, the sample was titrated, and the peroxide value (measured in meq O_2_/kg oil) was calculated as follows: PV = V × N/W 100, where V is the Na_2_S_2_O_3_ titration volume in milliliters and W is the sample weight in grams.

### 2.10 Fiber Content

The control and fortified crackers (nonencapsulated PE, EPEOP, and EPEP) were analyzed for total dietary fiber using the enzymatic‐gravimetric method according to AOAC (2000). All measurements were performed in triplicate, and results were expressed as a percentage on a dry weight basis.

### 2.11 Sensory Evaluation

A score sheet was used for the sensory evaluation. Control crackers and crackers fortified with nonencapsulated PE and encapsulated extracts (EPEOP and EPEP) were examined using the method described by (McCain‐Keefer et al. 2020). Qualified panelists (*n* = 10) from the Department of Nutrition and Food Science were asked to rate samples according to the scale: very good (9–10), good (7–8), moderate (5–6), poor (3–4), and very poor (1–2). Panelists received crackers after baking 24 h on plastic white plates with three‐digit codes generated randomly. Taste, odor, texture, color, and general acceptability were evaluated by a panel of individuals. The study was approved by the Research Ethics Committee of King Faisal University (Approval No. KFU‐REC‐ETHICS4344). All panelists participated voluntarily and provided informed consent prior to their participation in the sensory evaluation.

### 2.12 Statistical Analysis

Statistical analyses were performed using IBM SPSS Statistics version 23.0 (IBM Corp., Armonk, NY, USA). Data are presented as mean ± standard deviation (SD). Homogeneity of variance was assessed using Levene's test before analysis. Parametric data were analyzed using one‐way analysis of variance (ANOVA). Mean comparisons among treatments at each storage time and among storage times within each treatment were performed using Duncan's multiple range test at *p* < 0.05. Comparisons between two conditions, such as before and after baking, were performed using the independent‐samples *t*‐test. Sensory evaluation data were analyzed using the Kruskal–Wallis test. Statistical significance was set at *p* < 0.05.

## 3 Results and Discussion

### 3.1 Phenolic Compounds Identification in Nonencapsulated PE and Encapsulated Extracts (EPEOP and EPEP)

HPLC analysis revealed the presence of 17 phenolic compounds in the unencapsulated pomegranate peel extract (PE), while 18 and 16 compounds were identified in the encapsulated extracts EPEOP and EPEP, respectively (Table 1). Punicalagin, gallic acid, chlorogenic acid, catechin, caffeic acid, syringic acid, and ellagic acid represented the highest concentrations in pomegranate PE and its capsules. The results indicated that ferulic acid and quercetin concentrations were higher in the orange peel‐encapsulated extract (EPEOP) compared to the unencapsulated extract (PE) and pectin‐encapsulated extract (EPEP), while methyl gallate and naringenin concentrations were higher in the unencapsulated pomegranate (PE) compared to the encapsulated extracts.

**TABLE 1:** High‐performance liquid chromatography analysis of polyphenolic compounds in nonencapsulated PE and encapsulated extract (EPEOP and EPEP).

Compounds	PE		EPEOP		EPEP	
	Conc. (μg/mL = 20 mg/mL)	Conc. μg/g	Conc. (μg/mL = 20 mg/ml)	Conc. μg/g	Conc. (μg/mL = 20 mg/mL)	Conc. μg/g
Punicalagin	1250.30	62515.00	850.45	42522.50	780.20	39010.00
Gallic acid	997.45	49872.59	577.43	28871.30	579.34	28966.82
Chlorogenic acid	156.31	7815.44	106.66	5332.87	91.55	4577.61
Catechin	28.65	1432.31	16.37	818.42	15.35	767.40
Methyl gallate	3.71	185.56	1.08	53.91	1.35	67.54
Coffeic acid	8.82	441.19	5.68	283.99	6.03	301.64
Syringic acid	14.08	703.91	9.24	462.13	9.91	495.75
Rutin	N.D.	N.D.	0.22	11.09	0.22	11.10
Ellagic acid	83.88	4193.84	40.24	2012.04	42.18	2109.02
Coumaric acid	0.42	20.93	0.22	10.92	0.20	9.82
Vanillin	0.24	11.95	0.18	8.79	0.15	7.58
Ferulic acid	0.80	39.83	9.62	481.23	0.64	32.00
Naringenin	2.32	115.87	1.03	51.37	1.15	57.41
Rosmarinic acid	0.69	34.38	0.78	39.04	0.26	12.80
Daidzein	N.D.	N.D.	N.D.	N.D.	N.D.	N.D.
Querectin	0.97	48.44	11.31	565.68	0.24	11.91
Cinnamic acid	0.05	2.66	0.06	3.00	0.10	5.07
Kaempferol	0.13	6.60	0.17	8.65	N.D.	N.D.
Hesperetin	0.66	33.14	0.09	4.66	N.D.	N.D.

*Note:* HPLC data are presented descriptively and were not subjected to statistical analysis.

Abbreviations: Conc., concentration; EPEOP, encapsulated pomegranate extract in orange peel; EPEP, encapsulated pomegranate extract in pectin; N.D., not detected; PE, pomegranate extract.

The diverse phenolic profile observed in pomegranate peel extract aligns with previous findings by Man et al. (2022) and Yan et al. (2017), who reported punicalagin as the most abundant phenolic compound in pomegranate peels, followed by gallic acid as reported by Çam and İçyer (2015) and Singh et al. (2023). The variation in phenolic compound concentrations between unencapsulated and encapsulated extracts can be attributed to the effect of the encapsulation process on the extraction of these compounds from the carrier material. Previous studies have reported that orange peel contains significant amounts of ferulic acid and quercetin (Liu et al. 2025; Panić et al. 2021), which explains their higher concentrations in the orange peel‐encapsulated extract (EPEOP). Sanhueza et al. confirmed that the carrier material used in the encapsulation process can influence the phenolic composition of the encapsulated extract (Sanhueza et al. 2022).

The lower concentrations of methyl gallate and naringenin in the encapsulated extracts compared to the unencapsulated extract may be attributed to binding interactions between these compounds and the carrier materials (orange peel or pectin), or due to the effect of freezing on the stability of these compounds. According to Ledari et al. (2024), although lyophilization operates at low temperatures, certain phenolic compounds may be affected by ice crystal formation during freezing or may undergo oxidation during pre‐lyophilization preparation stages. Additionally, Fang and Bhandari reported that the molecular structure and polarity of specific phenolic compounds can influence their retention efficiency during the encapsulation process, with some compounds showing a stronger affinity for certain carrier materials than others (Fang and Bhandari 2010).

### 3.2 Color Analysis of Nonencapsulated PE and Encapsulated Extracts (EPEOP and EPEP)

**FIGURE 1:**
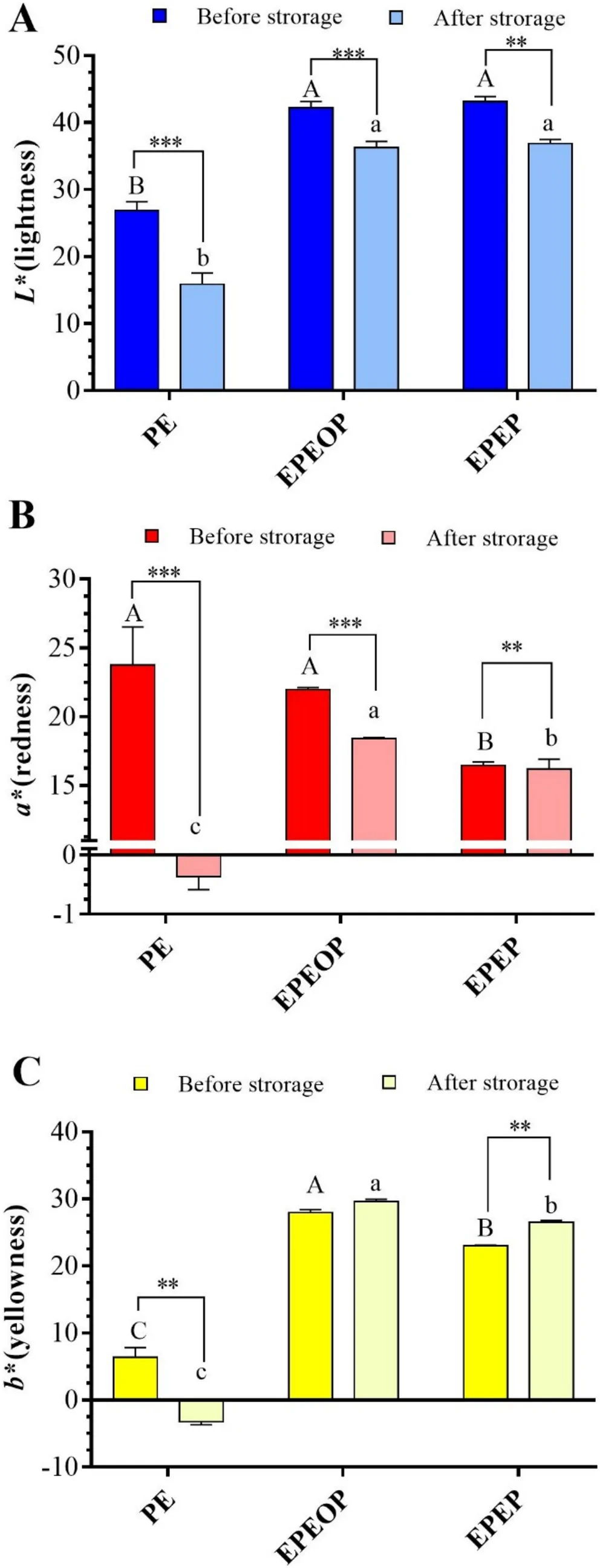
Color analysis of nonencapsulated PE and encapsulated extract (EPEOP and EPEP). PE, pomegranate extract; EPEOP, encapsulated pomegranate extract in orange peel; EPEP, encapsulated pomegranate extract in pectin. Capital letters (B and A) indicate differences between the treatments before storage. Lowercase letters (a and b) indicate differences between the treatments after storage. * Indicate differences between before and after storage or cooking within the treatment according to *t*‐test. Significant differences (*p* < 0.05) according to Duncan's post hoc test.

**FIGURE 2:**
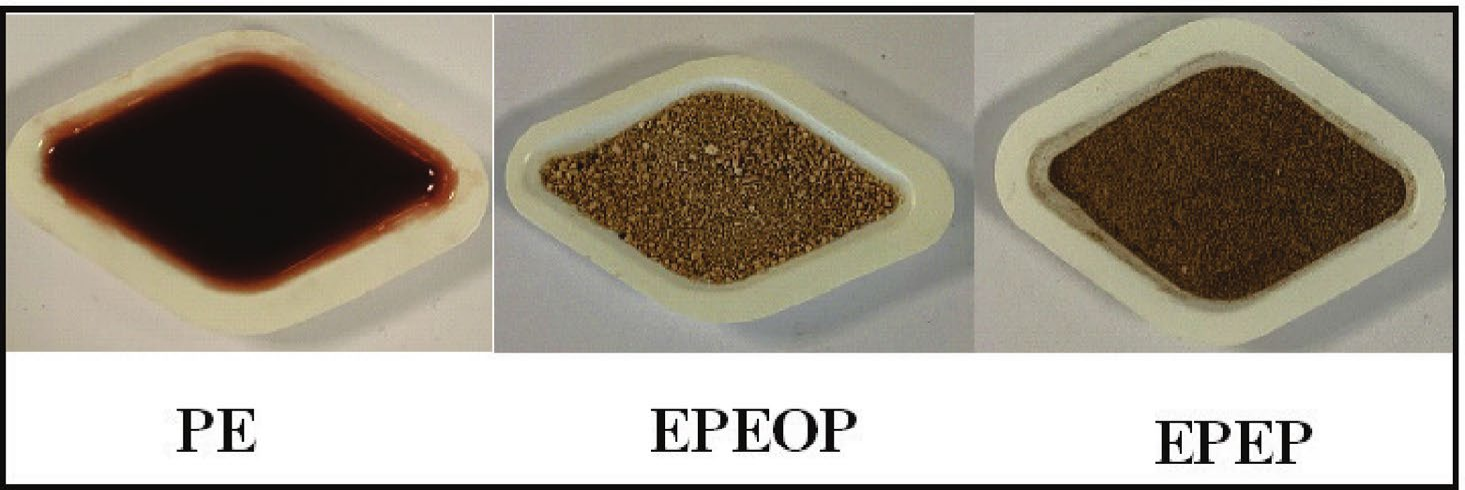
Color visualizations of nonencapsulated PE and encapsulated extract (EPEOP and EPEP).

Changes in color parameters a* (redness), b* (yellowness), and L* (lightness) of non‐encapsulated pomegranate peel extract (PE) and encapsulated extracts using orange peel (EPEOP) and pectin (EPEP) were evaluated before and after accelerated storage at 60°C for 45 days (Figure 1). Anthocyanins, particularly cyanidin‐3‐glucoside and delphinidin‐3‐glucoside, are the primary pigments responsible for the red coloration of pomegranate peel extracts (Gumienna et al. 2016). As shown in Figure 2, the non‐encapsulated extract exhibited a red color, whereas the encapsulated powders appeared more yellow due to the inherent color of the carrier materials.

For the redness parameter (*a*), a pronounced decline was observed in the non‐encapsulated extract after storage, with the a value decreasing from 23.77 to −0.37. In contrast, both encapsulated samples retained substantially higher redness, demonstrating the protective effect of encapsulation against pigment degradation. Similar color deterioration in non‐encapsulated pomegranate extracts has been attributed to anthocyanin degradation during storage (Rocha‐Parra et al. 2016; Cano‐Lamadrid et al. 2018). The improved stability observed in the encapsulated samples indicates that the encapsulation matrix acts as a protective barrier that limits exposure to oxygen and other environmental factors responsible for pigment degradation. Differences between the carrier materials may explain the observed stability patterns. Orange peel contains dietary fiber, pectin, and phenolic compounds that can stabilize anthocyanins through hydrogen bonding, copigmentation, and physical entrapment, thereby reducing their exposure to degradation (De Freitas et al. 2017; Jakobek 2015). In contrast, pectin stabilizes anthocyanins mainly through gel formation and electrostatic interactions, although its higher hydrophilicity may facilitate water penetration and pigment degradation during storage (Elwers et al. 2009). These mechanisms may contribute to the slightly better redness retention observed in EPEOP. However, because different encapsulation strategies were applied for the two wall materials, the present study cannot distinguish the individual contributions of the wall material and the encapsulation strategy.

Encapsulated samples exhibited significantly higher and more stable *b* values during storage than the non‐encapsulated extract, reflecting the inherent color of the carrier materials and their protective effect against pigment degradation. These findings are consistent with previous reports on encapsulated pomegranate extracts (Kaderides et al. 2020). Encapsulated samples also maintained significantly greater *L* stability than the non‐encapsulated extract during storage. The greater darkening observed in the non‐encapsulated extract may be attributed to polymerization of phenolic compounds and the formation of brown pigments during storage at elevated temperature. The improved color stability of the encapsulated samples may also be attributed to the lyophilization process, which preserves phenolic compounds by minimizing thermal degradation and promoting their physical entrapment within the carrier matrix (Ezhilarasi et al. 2013; Kaderides et al. 2020). Overall, encapsulation effectively preserved the color of pomegranate peel extract during storage, supporting the application of orange peel and pectin as natural carrier materials for color‐sensitive food systems (Chezanoglou et al. 2023).

### 3.3 Stability of Phenolic Antioxidant in Nonencapsulated PE and Encapsulated Extracts (EPEOP and EPEP) During Storage

#### 3.3.1 Change in Total Phenolic Content

**FIGURE 3:**
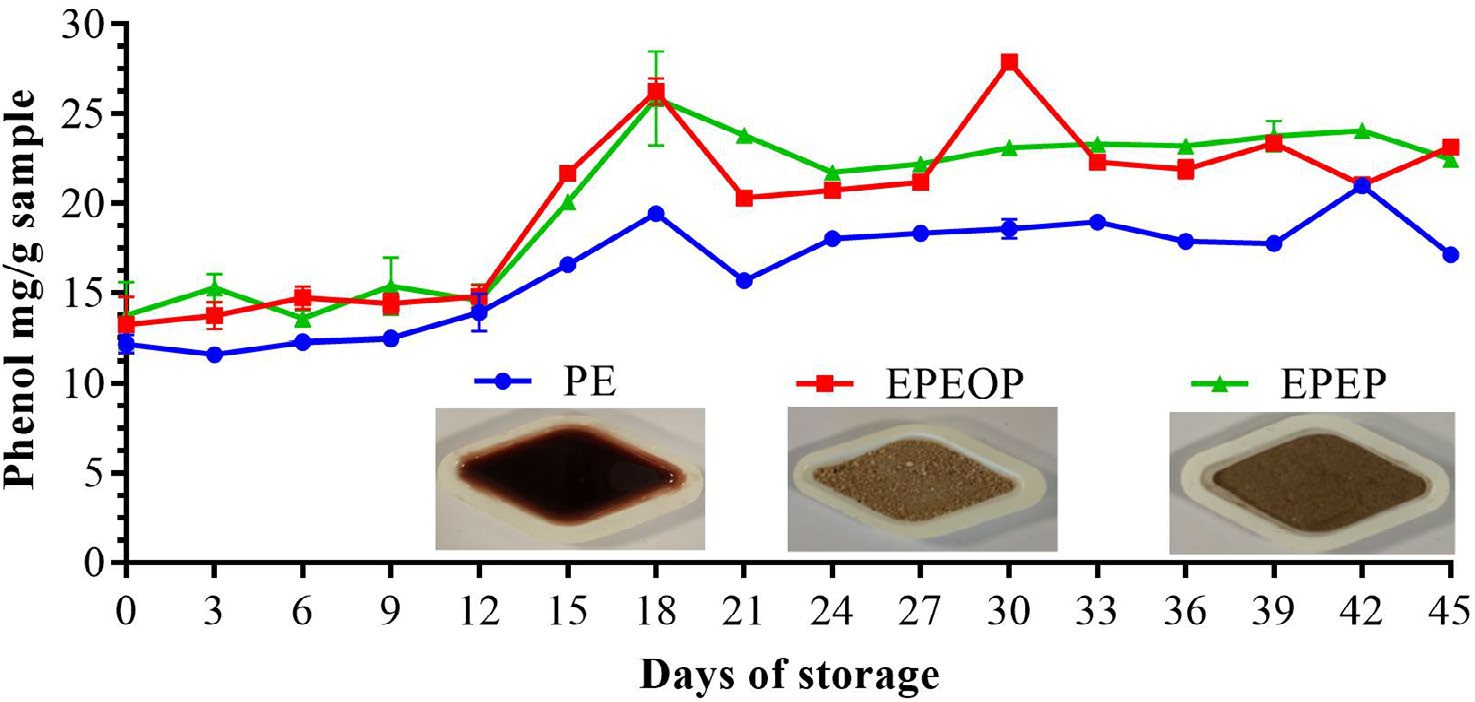
Total phenolics content of nonencapsulated PE and encapsulated extract (EPEOP and EPEP) during storage at 60°C. PE, pomegranate extract; EPEOP, encapsulated pomegranate extract in orange peel; EPEP, encapsulated pomegranate extract in pectin. Statistical comparisons among treatments at each storage time and among storage times within each treatment are presented in Table S1.

The total phenolic content (TPC) of the non‐encapsulated pomegranate peel extract (PE) and the encapsulated samples (EPEOP and EPEP) was monitored during 45 days of storage (Figure 3). No significant differences were observed among treatments at day 0; however, significant differences developed during storage, with both encapsulated systems generally maintaining higher TPC than the non‐encapsulated extract.

All treatments showed a progressive increase in total phenolic content (TPC) during the storage period, with both linear and quadratic trends being statistically significant (*p* < 0.001). The non‐encapsulated extract (PE) exhibited an increase of 40.5% in TPC from day 0 to day 45, whereas greater increases were observed for the encapsulated systems, reaching 75.0% for EPEOP and 63.0% for EPEP (Table S1). Although phenolic compounds are typically prone to degradation during storage as a result of oxidation and related reactions, an apparent increase in TPC has been reported in several phenolic‐rich systems (Chezanoglou et al. 2023). Comparable observations were documented in stored pomegranate fruits (Selcuk and Erkan 2015) and in encapsulated grape marc extracts under elevated temperature conditions (Moreno et al. 2018).

This increase in measured phenolic content may be explained by more than one mechanism. One likely explanation is the gradual release of bound phenolic compounds from complex structures within the extract matrix, which enhances their accessibility to the Folin–Ciocalteu reagent used in TPC determination (Fawole and Opara 2016). In addition, the hydrolysis of complex polyphenols into simpler phenolic forms during storage may also contribute to the observed increase in measurable TPC (Cao et al. 2021). Similar behavior has been reported for spray‐dried pomegranate peel extracts, where phenolic levels increased during the early stages of storage (Kaderides et al. 2020). The more pronounced increase observed after approximately 12 days of storage may indicate that sufficient time was required for structural modifications within the extract and encapsulation matrices to occur. Such structural changes may facilitate the gradual hydrolysis of phenolic–polysaccharide or phenolic–protein associations, leading to enhanced release of previously bound phenolic compounds and increased accessibility to the Folin–Ciocalteu reagent. Similar delayed increases in measurable phenolic content during storage have been attributed to progressive matrix relaxation and hydrolytic reactions in phenolic‐rich food systems (Fawole and Opara 2016; Cao et al. 2021).

The maximum TPC values were reached at different time points depending on the encapsulation system. EPEP reached its peak earlier, recording 25.86 ± 2.62 mg/g on day 18. In contrast, EPEOP exhibited a slower but more pronounced increase, reaching the highest TPC value among all treatments (27.88 ± 0.11 mg/g) on Day 30. The non‐encapsulated extract showed a more gradual pattern, reaching its maximum value of 20.98 ± 0.09 mg/g on Day 42. The largest difference was observed on Day 30, where EPEOP exhibited approximately 50% higher TPC than PE. These findings support the protective role of encapsulation in preserving phenolic compounds during storage. Similar outcomes have been reported in previous studies, where encapsulated systems demonstrated improved phenolic retention compared with non‐encapsulated counterparts (Yinbin et al. 2018; Xu et al. 2019). For instance, Chezanoglou et al. (2023) reported minimal changes in phenolic content in co‐crystallized pomegranate peel extracts stored at elevated temperatures, confirming the effectiveness of encapsulation in limiting thermal degradation.

Differences in stability behavior were also evident between the two encapsulation matrices. The earlier peak observed for EPEP may indicate a faster release of phenolic compounds from the pectin matrix, which can be attributed to its hydrophilic and gel‐forming characteristics. In contrast, the orange peel matrix appeared to provide a slower and more sustained release of phenolic compounds, which may explain the delayed peak observed for EPEOP. This could be related to the structural complexity of orange peel, which contains polysaccharides such as pectin, cellulose, and hemicellulose that can form a more compact matrix capable of entrapping phenolic compounds and reducing their exposure to degradation factors (Kaderides et al. 2020; Liu et al. 2025). Moreover, orange peel naturally contains antioxidant constituents, including flavonoids and vitamin C, which may further contribute to the stabilization of phenolic compounds by limiting oxidative reactions during storage. This may explain why EPEOP achieved the highest TPC values among all treatments. Overall, both encapsulation systems were effective in maintaining phenolic stability during storage. Although the orange peel system exhibited superior phenolic retention, the present study was not designed to distinguish whether this advantage resulted from the wall material itself or from differences in the encapsulation strategy. Therefore, the observed superiority should be interpreted as the combined effect of both factors.

#### 3.3.2 Change in Antioxidant Activity

**FIGURE 4:**
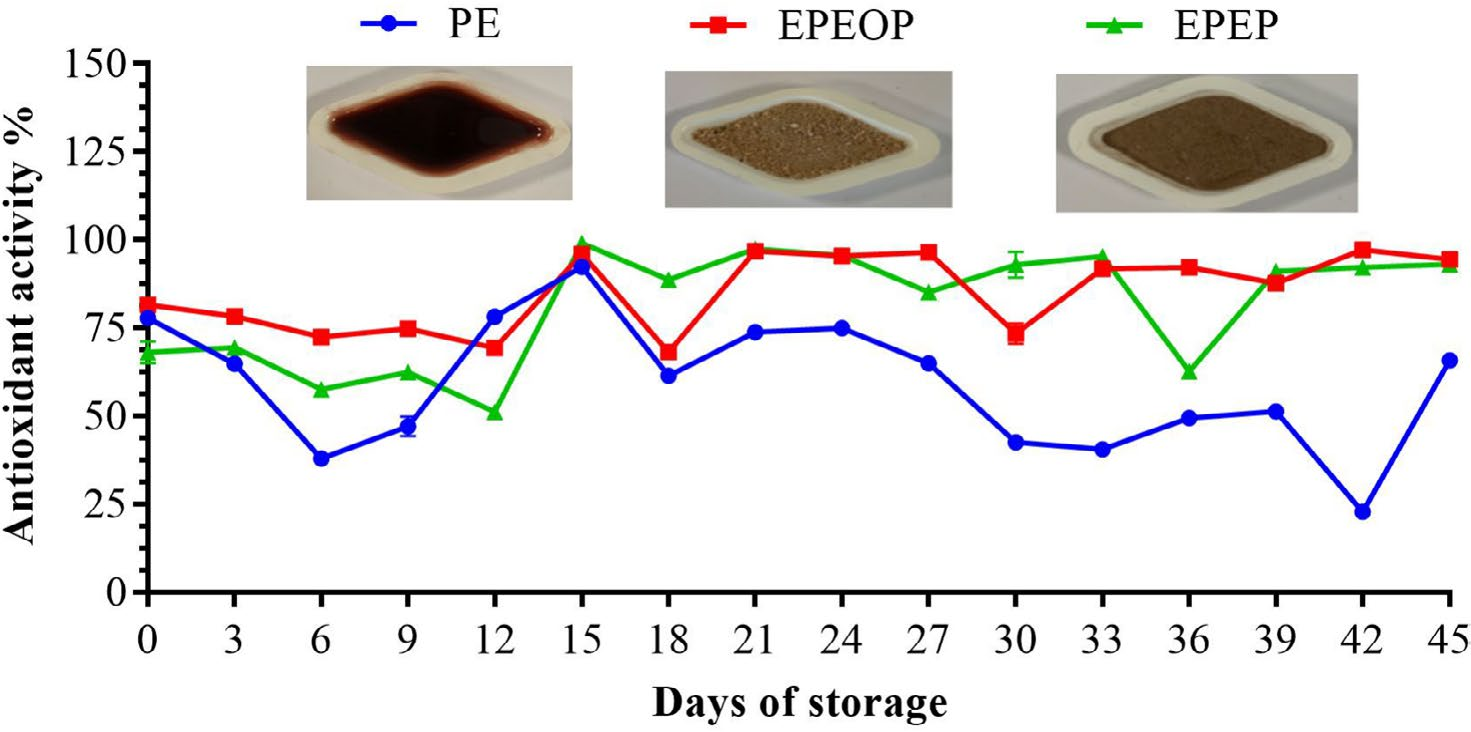
Antioxidant activity of nonencapsulated PE and encapsulated extract (EPEOP and EPEP) during storage at 60°C. PE, pomegranate extract; EPEOP, encapsulated pomegranate extract in orange peel; EPEP, encapsulated pomegranate extract in pectin. Statistical comparisons among treatments at each storage time and among storage times within each treatment are presented in Table S2.

Figure 4 presents the antioxidant activity of PE, EPEOP, and EPEP during 45 days of storage. Significant differences were observed among treatments and throughout the storage period (*p* < 0.001 for treatment, linear, and quadratic effects).

At Day 0, EPEOP exhibited the highest antioxidant activity (81.53% ± 1.04%), followed by PE (77.80% ± 0.37%) and EPEP (68.03% ± 3.10%). Statistical analysis showed significantly higher antioxidant activity in EPEOP and PE than in EPEP. This suggests that the encapsulation process, particularly with pectin, might initially impact the immediate accessibility or activity of some antioxidant compounds, or that the orange peel matrix inherently contributes to higher initial antioxidant capacity.

Throughout the 45‐day storage period, the antioxidant activity of the non‐encapsulated PE fluctuated considerably and exhibited an overall declining trend, reaching its lowest point at Day 42 (22.82% ± 1.09%) before a slight recovery. This significant decline in antioxidant activity for the unencapsulated extract is consistent with the general susceptibility of phenolic compounds to degradation when exposed to environmental factors such as oxygen, light, and heat (Tsali and Goula 2018). The degradation of these compounds leads to a reduction in their radical scavenging capacity.

In contrast, both encapsulated extracts (EPEOP and EPEP) demonstrated significantly superior stability in antioxidant activity compared to the non‐encapsulated PE (*p* < 0.001). EPEOP consistently maintained high antioxidant activity, with values generally above 90% from Day 21 to 45, peaking at 97.14% ± 0.28% on Day 42. Similarly, EPEP also exhibited remarkable stability, with activity frequently above 90%, reaching its highest point at 99.07% ± 0.19% on Day 15 and maintaining high levels thereafter (Table S2). The encapsulated extracts exhibited greater stability in antioxidant activity throughout storage. EPEOP showed a significant linear trend, whereas EPEP exhibited a significant quadratic trend (*p* < 0.001). These findings underscore the protective effect of the encapsulation matrices. The protective matrices of orange peel and pectin effectively shielded the encapsulated phenolic compounds from external degradative factors, thereby preserving their antioxidant potential. Similar observations were reported by Kaderides et al. (2020), who found that encapsulated pomegranate peel extract maintained high antioxidant activity during storage at 60°C, whereas the crude extract showed a marked decline. Chezanoglou et al. also confirmed that encapsulation of pomegranate peel extract using the co‐crystallization technique improved the stability of antioxidant activity (Chezanoglou et al. 2023).

The slight fluctuations in antioxidant activity observed during storage may reflect changes in the phenolic profile resulting from the transformation of phenolic compounds into products with different antioxidant capacities. Therefore, antioxidant activity does not necessarily correlate directly with total phenolic content. Moser et al. (2017) and Rocha‐Parra et al. (2016) similarly found a lack of correlation between the loss of total anthocyanin or phenolic content and antioxidant capacity during the storage of microencapsulated red grape juice and encapsulated red wine, respectively (Moser et al. 2017; Rocha‐Parra et al. 2016). They attributed this trend to the formation of polymers, such as procyanidins, or the formation of degradation products of polyphenols with high antioxidant activity.

The superior performance of the encapsulated extracts, particularly EPEOP, in maintaining antioxidant activity is a crucial finding. The orange peel matrix, rich in dietary fiber, natural pectin, and other phenolic compounds, likely provides a robust protective environment. The cellulosic fiber matrix in orange peel offers physical entrapment, limiting the mobility and exposure of antioxidant molecules to degradative factors (Robert et al. 2015). Pectin, on the other hand, forms a hydrophilic gel network that can entrap these compounds through hydrogen bonding and hydrophobic interaction (Maier et al. 2009). The gentle lyophilization process employed in this study further contributes to this stability by preserving the native structure of both the carrier materials and the bioactive compounds, allowing optimal molecular interactions and the formation of a glassy matrix that immobilizes reactive species (Ciurzyńska and Lenart 2011).

### 3.4 Stability of Phenolic Antioxidant in Crackers During the Baking Process

**FIGURE 5:**
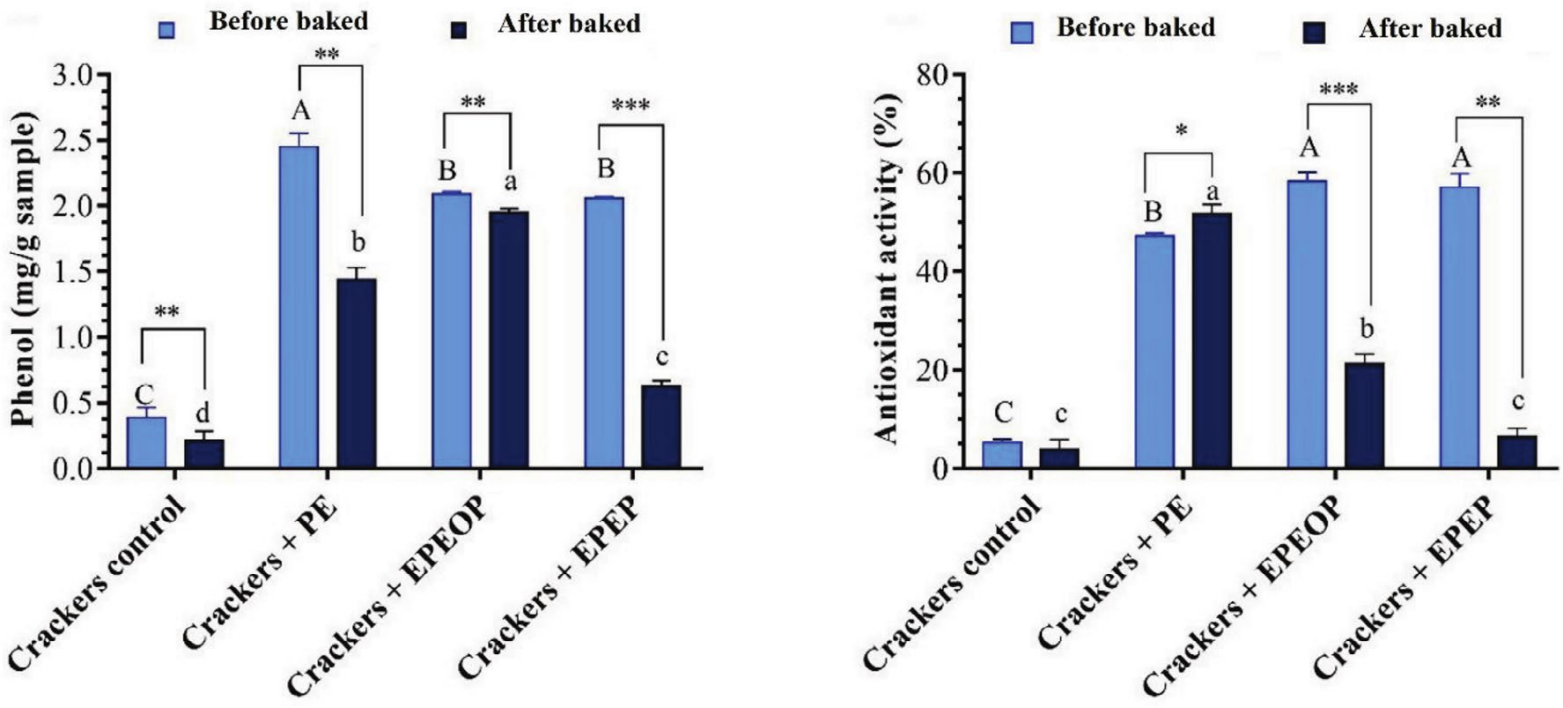
Total phenol content and antioxidant activity (%) for crackers enriched with nonencapsulated PE and encapsulated extract (EPEOP and EPEP) before and after baking. PE, pomegranate extract; EPEOP, encapsulated pomegranate extract in orange peel; EPEP, encapsulated pomegranate extract in pectin. Capital letters (B and A) indicate differences between treatments before baking. Lowercase letters (a, b) indicate differences between treatments after baking. * Indicate differences between before and after baking within the treatment according to the independent‐samples *t*‐test. Significant differences were determined according to Duncan's post hoc test (*p* < 0.05).

The stability of unencapsulated pomegranate peel extract (PE) and encapsulated extracts (EPEOP and EPEP) during the baking process was evaluated by measuring the TPC and antioxidant activity before and after baking crackers, as shown in Figure 5. The results showed a significant decrease (*p* < 0.05) in TPC and antioxidant activity for all extracts after the baking process. Before baking, crackers fortified with unencapsulated extract (PE) recorded the highest TPC (2.45 ± 0.10 mg/g), followed by cookies fortified with orange peel encapsulated extract (EPEOP) (2.09 ± 0.02 mg/g), then cookies fortified with pectin encapsulated extract (EPEP) (2.06 ± 0.01 mg/g). The lowest value was recorded in unfortified crackers. After baking, the TPC decreased by varying percentages, with the lowest decrease in crackers fortified with orange peel‐encapsulated extract (EPEOP) (6.7%), followed by cookies fortified with unencapsulated extract (PE) (40.8%), then crackers fortified with pectin‐encapsulated extract (EPEP) (69.4%). On the other hand, the sample of crackers fortified with orange peel capsules remains the highest significant among the fortified and control samples.

As for antioxidant activity, crackers fortified with orange peel‐encapsulated extract (EPEOP) recorded the highest antioxidant activity before baking (58.46% ± 1.79%), followed by cookies fortified with pectin‐encapsulated extract (EPEP) (57.21% ± 2.72%), then cookies fortified with unencapsulated extract (PE) (47.33% ± 0.54%). After baking, the antioxidant activity of cookies fortified with unencapsulated extract (PE) increased to 51.80% ± 1.81%, while it significantly decreased in cookies fortified with encapsulated extracts EPEOP and EPEP to reach 21.27% ± 2.05% and 6.53% ± 1.71%, respectively. No significant differences were observed between cookies fortified with encapsulated extract EPEP and the control. The baking process led to a significant decrease in TPC and antioxidant activity for all extracts. This is likely due to thermal processing enhancing the interaction of polyphenols with other cookie components, including sugar fragments produced through caramelization and Maillard reactions (Tumbas Šaponjac et al. 2016). In addition, the decrease observed in TPC and antioxidant activity for all extracts after baking was of varying degrees. The orange peel encapsulated extract (EPEOP) showed the best stability of TPC during the baking process, with only a 6.7% decrease, compared to 40.8% and 69.4% for the unencapsulated extract (PE) and pectin‐encapsulated extract (EPEP), respectively.

These results are consistent with those of Kaderides et al., who reported that a significant amount of phenolic compounds degrade during the baking process even if they are encapsulated, but encapsulation has a significant effect on retaining phenolic compounds and their activity compared to the unencapsulated extract (Kaderides et al. 2020). Abdel‐Aal et al. also confirmed that the baking process leads to a decrease in bound phenolic acids, while free phenolic acids increase (Abdel‐Aal and Rabalski 2013). According to Dueik & Bouchon, frying starchy matrices supplemented with olive leaf phenolic extract at 160°C resulted in a 42% retention of polyphenols (Dueik and Bouchon 2016). Luca et al. observed even lower values, with cakes enriched with crude and encapsulated sour cherry phenolics retaining 22.2% and 30.7% of their total phenolic content, respectively, after baking (Luca et al. 2014). Likewise, Saponjac et al. reported a 43% reduction in phenolic compounds in cookies fortified with encapsulated cherry pomace extract following baking at 230 C for 15 min (Tumbas Šaponjac et al. 2016).

The difference in stability of phenolic compounds between the different extracts during the baking process can be explained by the effect of the carrier material in protecting these compounds from thermal effects. The orange peel‐encapsulated extract (EPEOP) showed better stability of TPC during the baking process, which may be due to the high fiber content of orange peel that may provide physical protection for phenolic compounds from thermal effects. Žilić et al. (2016) reported that the stability of phenolic compounds during the baking process is affected by several factors, including temperature, baking time, and dough composition (Žilić et al. 2016). As for antioxidant activity, cookies fortified with unencapsulated extract (PE) showed an increase in antioxidant activity after baking, while antioxidant activity significantly decreased in cookies fortified with encapsulated extracts. This increase in antioxidant activity of cookies fortified with unencapsulated extract can be explained by the formation of Maillard reaction products during the baking process, which may possess antioxidant activity. The observed discrepancy between the high retention of TPC and the significant decline in antioxidant activity for EPEOP can be attributed to the physical protection provided by the orange peel fiber matrix. Although encapsulation effectively protected phenolic compounds from thermal degradation, it may also have limited their release and accessibility during antioxidant determination, resulting in lower measured radical‐scavenging activity despite the high retention of total phenolics (Tolve et al. 2016; Kaderides et al. 2020). Conversely, the increased antioxidant activity in crackers fortified with non‐encapsulated PE, despite higher phenolic loss, is primarily linked to the intensive formation of Maillard reaction products (MRPs), such as melanoidins, which exhibit potent radical scavenging properties (Žilić et al. 2016). Furthermore, the baking process has been reported to decrease bound phenolic acids while increasing free phenolic acids, further contributing to the enhanced antioxidant potential of the non‐encapsulated extract (Abdel‐Aal and Rabalski 2013). Encapsulation may have restricted the release and accessibility of polyphenols from the carrier matrix during baking and subsequent antioxidant determination, which may partly explain the lower antioxidant activity observed in the encapsulated samples despite their higher phenolic retention (Šaponjac et al. 2017). Žilić et al. (2016) confirmed that Maillard reaction products formed during the baking process may affect the antioxidant activity of the final product (Žilić et al. 2016). Similarly, Tolve et al. (2022) reported that encapsulation improves the thermal stability of polyphenols during baking; however, the extent to which antioxidant activity is retained depends on the encapsulating material, food matrix, and release behavior of the encapsulated compounds.

### 3.5 Stability of Phenolic Antioxidants in Crackers During Storage

#### 3.5.1 Change in Total Phenolic Content

**FIGURE 6:**
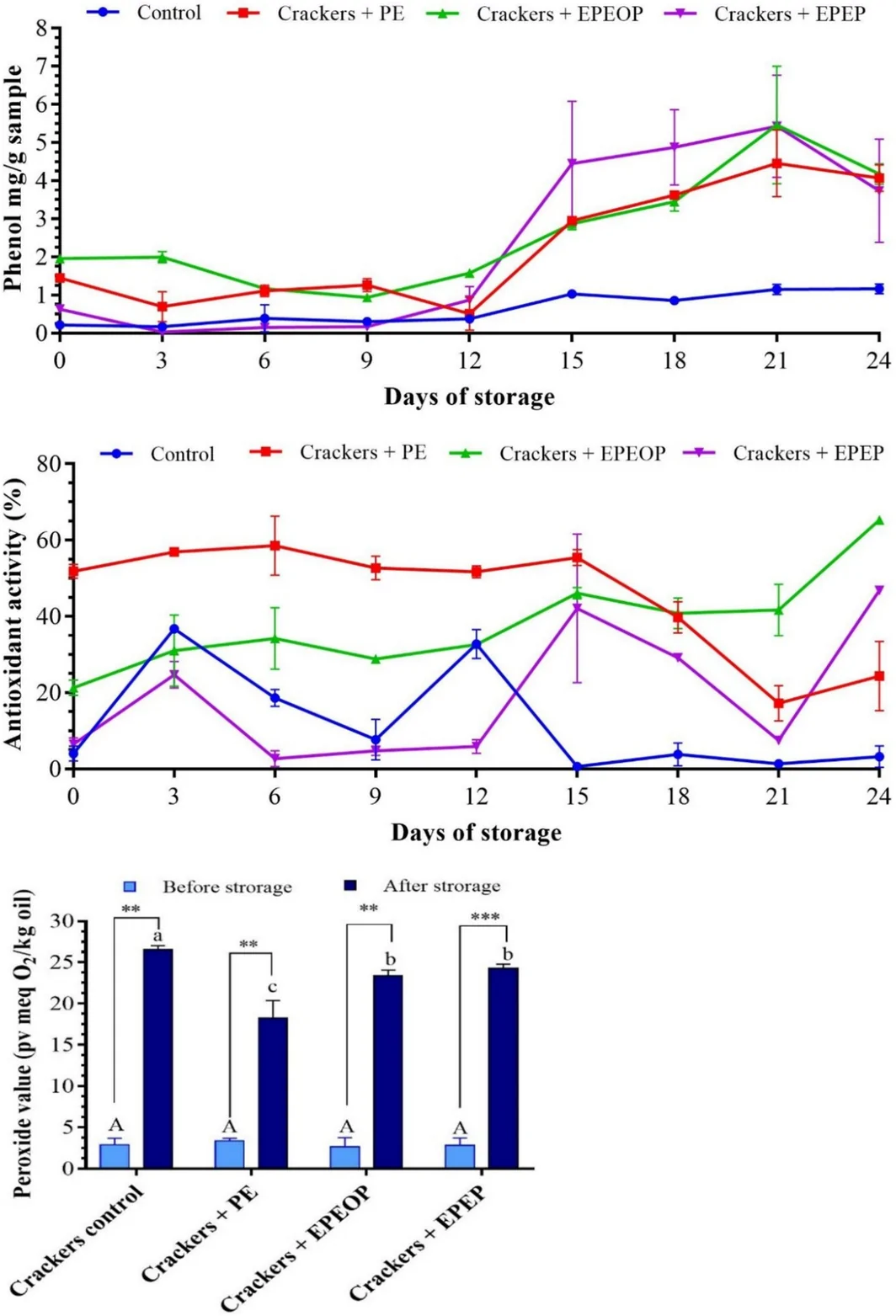
Total phenol content, antioxidant activity (%), and peroxide value for crackers enriched with nonencapsulated PE and encapsulated extract (EPEOP and EPEP) during storage at 60°C. PE, pomegranate extract; EPEOP, encapsulated pomegranate extract in orange peel; EPEP, encapsulated pomegranate extract in pectin. Detailed statistical comparisons among treatments at each storage time and among storage times within each treatment for total phenolic content and antioxidant activity are presented in Tables S3 and S4, respectively. For the peroxide value data, capital letters (B and A) indicate differences between treatments before storage. Lowercase letters (a & b) indicate differences between treatments after storage. * Indicate differences between before and after storage within the same treatment according to the independent‐samples *t*‐test. Significant differences were determined according to Duncan's post hoc test (*p* < 0.05).

Figure 6 shows the changes in total phenolic content (TPC) of crackers during 24 days of storage. Statistical analysis indicated significant effects of treatment and storage time (*p* < 0.001) (Table S3). At Day 0, all fortified crackers contained significantly higher TPC than the control (*p* < 0.001), confirming the successful incorporation of pomegranate peel phenolics into the cracker matrix. Among the fortified formulations, EPEOP exhibited the highest initial TPC. This may reflect the contribution of phenolic compounds naturally present in orange peel in addition to the encapsulated extract (Andrade et al. 2022). During storage, TPC increased in all formulations, with the encapsulated treatments consistently maintaining higher values than the non‐encapsulated extract. An increase in measured phenolic content during storage has been reported in several food systems. One possible explanation is the gradual degradation of complex phenolic structures into smaller compounds that react more readily with the Folin–Ciocalteu reagent (Choi et al. 2011). In addition, products generated through Maillard reactions during baking and subsequent storage may contribute to the measured phenolic values because of their reducing properties (Tang et al. 2024). Another factor may be the progressive release of phenolic compounds that are initially bound within the food matrix or entrapped within the encapsulation system (Abdel‐Aal and Rabalski 2013).

Encapsulation played an important role in maintaining phenolic stability during storage. Crackers fortified with encapsulated extracts consistently exhibited higher TPC than those containing the non‐encapsulated extract, with EPEOP showing the greatest phenolic stability throughout storage. The improved stability observed in encapsulated samples can be attributed to the protective barrier provided by the wall materials, which may limit the exposure of phenolic compounds to oxygen, light, and moisture during storage (Grgić et al. 2020). The slightly higher stability observed for the orange peel system may be related to its complex composition. Orange peel contains dietary fiber, natural pectin, and endogenous antioxidants, which may enhance the protection of phenolic compounds and reduce their degradation during storage (Andrade et al. 2022). Such interactions could contribute to the improved retention of phenolic compounds in EPEOP compared with the pectin‐based system. In contrast, crackers containing non‐encapsulated extract showed lower phenolic levels and greater variability during storage, suggesting that phenolic compounds were more susceptible to degradation in the absence of a protective matrix. Similar observations have been reported in bakery products fortified with encapsulated plant extracts, where encapsulation improved phenolic retention and antioxidant stability during storage (Bińkowska et al. 2024). Likewise, Tumbas Šaponjac et al. (2016) reported higher polyphenol retention in cookies enriched with encapsulated sour cherry pomace extract compared with control samples during storage. Overall, the addition of pomegranate peel extract increased the phenolic content of crackers, while encapsulation improved the stability of these compounds during storage. Among the tested systems, the orange peel‐based encapsulation system showed promising potential for maintaining phenolic antioxidants in baked products. However, because orange peel was evaluated using a two‐stage lyophilization process whereas commercial pectin was prepared using a conventional single‐stage process, the observed superiority should be interpreted as the combined effect of both the wall material and the encapsulation strategy.

#### 3.5.2 Change in Antioxidant Activity

Figure 6 shows the antioxidant activity of the cracker samples during 24 days of storage. The comparison included unfortified crackers (control), crackers fortified with non‐encapsulated pomegranate peel extract (Crackers +PE), and crackers fortified with encapsulated extract using orange peel (Crackers +EPEOP) or pectin (Crackers +EPEP). Statistical analysis revealed significant effects of treatment and storage time (*p* < 0.001) (Table S4). At Day 0, Crackers +PE exhibited the highest antioxidant activity, whereas the encapsulated formulations showed lower initial activity than the non‐encapsulated extract. The higher initial activity observed in Crackers +PE indicates the immediate availability of antioxidant compounds when the extract is directly incorporated into the cracker matrix. In contrast, the lower initial activity of the encapsulated formulations may be attributed to the formation of a protective matrix around the phenolic compounds, which limits their immediate interaction with the DPPH radical and delays their release (Grgić et al. 2020).

During storage, the antioxidant activity of the different formulations followed distinct trends. The non‐encapsulated extract showed relatively high activity during the early storage period, followed by a gradual decline, whereas both encapsulated formulations exhibited greater stability and progressively increased antioxidant activity throughout storage. Among them, EPEOP showed the highest antioxidant activity at the end of storage, while the control consistently exhibited the lowest values. This improved stability can be attributed to the protective role of the encapsulation matrix, which reduces the exposure of phenolic compounds to environmental factors such as oxygen, light, and moisture that promote oxidative degradation (Bińkowska et al. 2024). Among the encapsulated systems, the orange peel matrix provided greater protection, likely because of its fiber network and naturally occurring antioxidant compounds, which may enhance the stabilization of phenolic compounds during storage (Andrade et al. 2022).

The gradual increase in antioxidant activity observed in some samples during storage may also be associated with chemical changes occurring within the food matrix. As discussed previously in Section 3.5.1, the degradation of complex phenolic compounds into smaller molecules can increase their reactivity with the DPPH reagent (Tang et al. 2024). In addition, Maillard reaction products formed during baking and storage may contribute to the measured antioxidant capacity due to their reducing properties (Abdel‐Aal and Rabalski 2022). The encapsulation matrix may further regulate the release of phenolic compounds over time, resulting in a more sustained antioxidant response. The fluctuations observed in antioxidant activity, particularly in the control and EPEP‐fortified crackers, may reflect changes in the composition and availability of antioxidant compounds during storage. Such variations may result from the simultaneous degradation of naturally occurring antioxidant compounds and the formation or release of other antioxidant‐active compounds, leading to temporary increases or decreases in the measured antioxidant activity. In EPEP‐fortified crackers, the gradual transformation and increased accessibility of encapsulated phenolic compounds during storage may further contribute to these fluctuations. Similar inconsistencies between total phenolic content and antioxidant activity during storage have been previously reported in phenolic‐rich food systems, where changes in phenolic composition and degradation products influenced the measured antioxidant capacity (Moser et al. 2017; Rocha‐Parra et al. 2016).

In contrast, crackers containing non‐encapsulated extract showed a clearer decline in antioxidant activity during storage, indicating that phenolic compounds were more susceptible to degradation in the absence of a protective barrier. Similar observations have been reported in baked products enriched with encapsulated plant extracts, where encapsulation improved the stability and retention of antioxidant compounds during storage (Costa et al. 2025; Ghasemi et al. 2017). Overall, fortification with pomegranate peel extract increased the antioxidant activity of crackers, while encapsulation contributed to improved stability during storage. Among the tested systems, the orange peel‐based encapsulation system demonstrated the most consistent preservation of antioxidant activity under the conditions evaluated in the present study. However, because the orange peel and pectin systems were prepared using different encapsulation strategies, this superiority should be interpreted as the combined effect of both the wall material and the encapsulation strategy.

### 3.6 Stability of Lipid Oxidation in Crackers During Storage

Figure 6 presents the peroxide value (PV) of the different cracker formulations before storage and after 24 days, which reflects the extent of lipid oxidation. The formulations included unfortified crackers (Crackers Control), crackers fortified with non‐encapsulated pomegranate extract (Crackers +PE), and crackers fortified with encapsulated extract using orange peel (Crackers +EPEOP) or pectin (Crackers +EPEP). At the beginning of storage, PV values ranged from 2.63 ± 1.14 to 3.39 ± 0.29 meq O_2_/kg oil, and no significant differences were observed among treatments (*p* = 0.726). These relatively low values indicate that the baking process did not substantially promote the formation of primary oxidation products, or that the antioxidants naturally present in the ingredients, including those from the added extracts, may have limited early oxidative reactions.

After 24 days of accelerated storage, PV increased significantly in all samples (*p* < 0.001), confirming the progression of lipid oxidation during storage. The control crackers exhibited the highest PV (26.54 ± 0.48 meq O_2_/kg oil). This increase is expected for lipid‐containing baked products, where storage conditions favor the formation of hydroperoxides as primary oxidation products (Maire et al. 2013). The addition of pomegranate extract clearly influenced oxidative stability. Crackers +PE showed a PV of 18.23 ± 2.15 meq O_2_/kg oil after storage, which was significantly lower than the control (*p* < 0.001). Crackers fortified with encapsulated extract also showed reduced oxidation compared with the control, with PV values of 23.36 ± 0.70 meq O_2_/kg oil for Crackers +EPEOP and 24.27 ± 0.52 meq O_2_/kg oil for Crackers +EPEP. These results indicate that pomegranate phenolics contributed to limiting oxidative reactions during storage. Crackers fortified with the non‐encapsulated extract exhibited the lowest PV among the fortified samples. This observation may be associated with the greater immediate availability of free phenolic compounds in the food matrix, allowing earlier interaction with lipid radicals. In contrast, encapsulated phenolics are expected to be released more gradually from the wall matrix, which may delay their immediate antioxidant action.

Although fortification reduced peroxide values compared with the control crackers, PV levels in all samples remained higher than the commonly reported limit of 10 meq O_2_/kg oil. This result is most likely related to the accelerated storage conditions applied in this study. Storage at elevated temperature (60°C) is known to intensify oxidation reactions, which can lead to faster accumulation of hydroperoxides even in the presence of antioxidant compounds. The ability of pomegranate phenolics to inhibit lipid oxidation has been widely attributed to several mechanisms, including free radical scavenging, metal chelation, and interference with pro‐oxidant reactions (Benchagra et al. 2021; Costa et al. 2025). Comparable protective effects of plant‐derived antioxidants in food systems have also been reported in previous studies (Das et al. 2021; Kandylis and Kokkinomagoulos 2020; Mousumi et al. 2025). Although crackers fortified with encapsulated extracts exhibited significantly lower peroxide values than the control, the non‐encapsulated extract resulted in the lowest peroxide value after storage. Therefore, under the accelerated storage conditions applied in the present study, the peroxide value data do not demonstrate a superior inhibitory effect of encapsulation on primary lipid oxidation. Nevertheless, encapsulation significantly improved the retention of total phenolic content and antioxidant activity during storage, as demonstrated in Sections 3.5.1 and 3.5.2. Therefore, under the experimental conditions of the present study, its principal advantage was preserving phenolic stability rather than providing greater inhibition of primary lipid oxidation. It should also be noted that peroxide value reflects only primary oxidation products, whereas secondary oxidation products are mainly responsible for rancid flavors and undesirable odors. Therefore, further evaluation of secondary oxidation markers would provide a more comprehensive assessment of oxidative stability.

### 3.7 Color Analysis in Crackers During Storage

**FIGURE 7:**
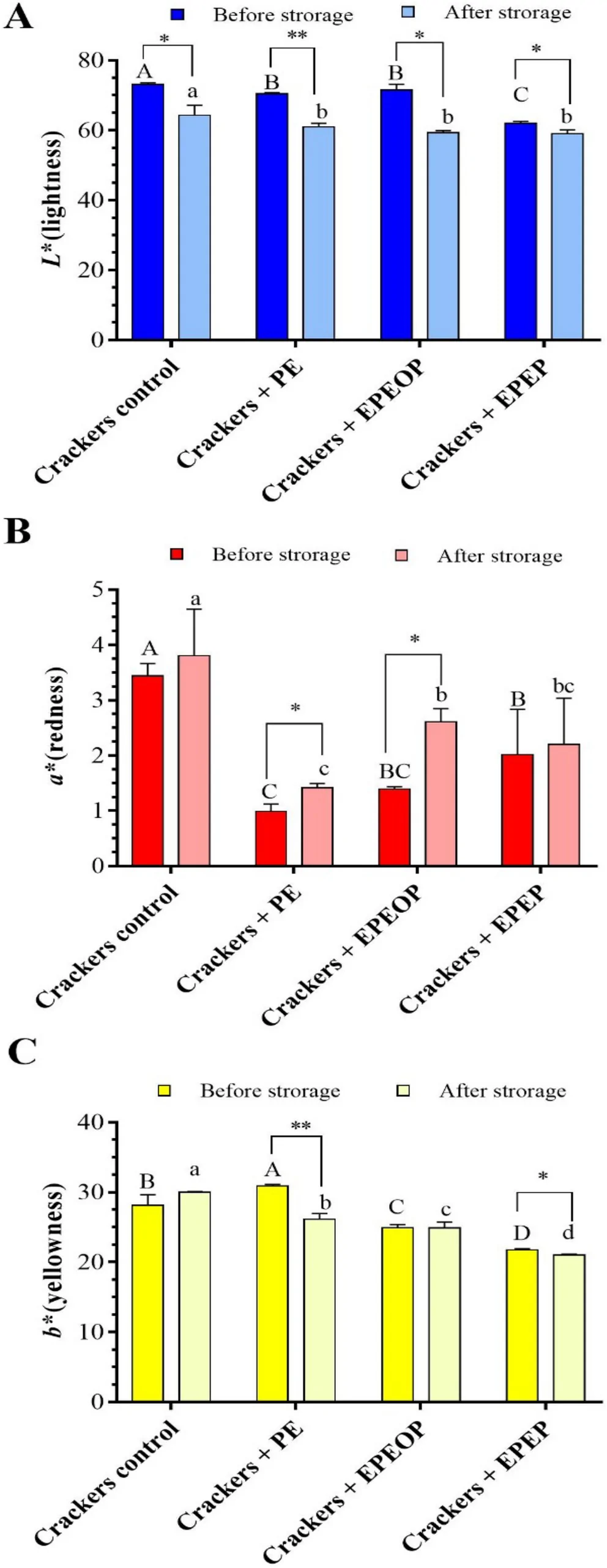
Color for crackers enriched with nonencapsulated PE and encapsulated extract (EPEOP and EPEP) before and after storage at 60°C, PE, pomegranate extract; EPEOP, encapsulated pomegranate extract in orange peel; EPEP, encapsulated pomegranate extract in pectin. Capital letters (B and A) indicate differences between the treatment before cooking. Lowercase letters (a, b) indicate differences between the treatments after cooking. * Indicate differences between before and after storage or cooking within the treatment according to the independent‐samples t‐test. Significant differences were determined according to Duncan's post hoc test (*p* < 0.05).

Figure 7 presents the color attributes (L* for lightness, a* for redness/greenness, and b* for yellowness/blueness) of fortified crackers before and after a 24‐day storage period. The results indicate significant changes in color attributes for all cracker formulations during storage, with varying degrees of stability influenced by fortification and encapsulation. Before storage, the control crackers exhibited the highest lightness (L*), whereas Crackers +EPEOP showed the lowest value. This suggests that the incorporation of pomegranate extract, especially the encapsulated forms, generally led to a darker product, with EPEOP resulting in the darkest crackers. This is likely due to the inherent color of the extracts and the encapsulating materials, as well as potential Maillard reactions during baking (Różańska et al. 2021).

After 24 days of storage, lightness decreased significantly in all formulations, indicating progressive darkening. The darkening during storage can be attributed to continued non‐enzymatic browning reactions, such as Maillard reactions and caramelization, which are accelerated by factors like moisture content and storage temperature (Manley 2011; Schutte et al. 2024). While all samples darkened, the encapsulated forms, particularly Crackers +EPEOP and Crackers +EPEP, maintained relatively consistent L* values compared to their initial state, suggesting that encapsulation might mitigate the extent of darkening over time. This is consistent with the protective effect of encapsulation against degradation processes, as discussed in earlier sections (e.g., Section 3.5.1 on TPC stability).

Before storage, redness (a*) varied significantly among formulations, with the control showing the highest value and Crackers +PE the lowest. This initial variation in redness is likely influenced by the color of the pomegranate extract and the encapsulating materials. Pomegranate extract itself can contribute to reddish tones due to anthocyanins, but the baking process and interactions with other ingredients can alter these pigments (Kaderides et al. 2020).

After storage, redness increased significantly in Crackers +PE and Crackers +EPEOP, whereas no significant changes were observed in the control and Crackers +EPEP. The increase in redness in some fortified samples during storage could be attributed to the formation of new red‐brown pigments through Maillard reactions or the oxidation of certain phenolic compounds (Tumbas Šaponjac et al. 2016). The encapsulated samples, particularly Crackers +EPEOP, demonstrated a more pronounced increase in redness, suggesting that the encapsulation matrix might influence the formation or stability of these color‐contributing compounds differently than in the non‐encapsulated or control samples.

Before storage, Crackers +PE exhibited the highest yellowness (b*), whereas Crackers +EPEP showed the lowest value. The higher yellowness in Crackers +PE and Crackers Control might be due to the natural pigments in the flour and butter, as well as Maillard reaction products. The lower yellowness in encapsulated samples, especially EPEP, could be due to the diluting effect of the encapsulating material or interactions that reduce the intensity of yellow pigments. After storage, no significant changes in yellowness were observed in the control or Crackers +EPEOP, whereas significant decreases occurred in Crackers +PE and Crackers +EPEP. The stability of yellowness in Crackers +EPEOP suggests that the orange peel matrix effectively protected the yellow‐contributing compounds, possibly due to its own carotenoid content and protective properties (Liu et al. 2025). This finding aligns with the results of Kaderides et al. (2020), who reported that the b* value remained unchanged following the incorporation of PE extracts into cookies. Furthermore, all color parameters showed minimal variation throughout the storage period. In conclusion, the color analysis of fortified crackers during storage revealed complex changes influenced by both fortification and encapsulation. While all samples showed a general darkening, the encapsulated forms, particularly those with orange peel, demonstrated better stability in terms of yellowness and a more controlled change in redness. These findings underscore the importance of selecting appropriate encapsulation materials to maintain the visual appeal and quality of fortified baked goods over their shelf‐life.

### 3.8 Fiber Content in Fortified Crackers

**FIGURE 8:**
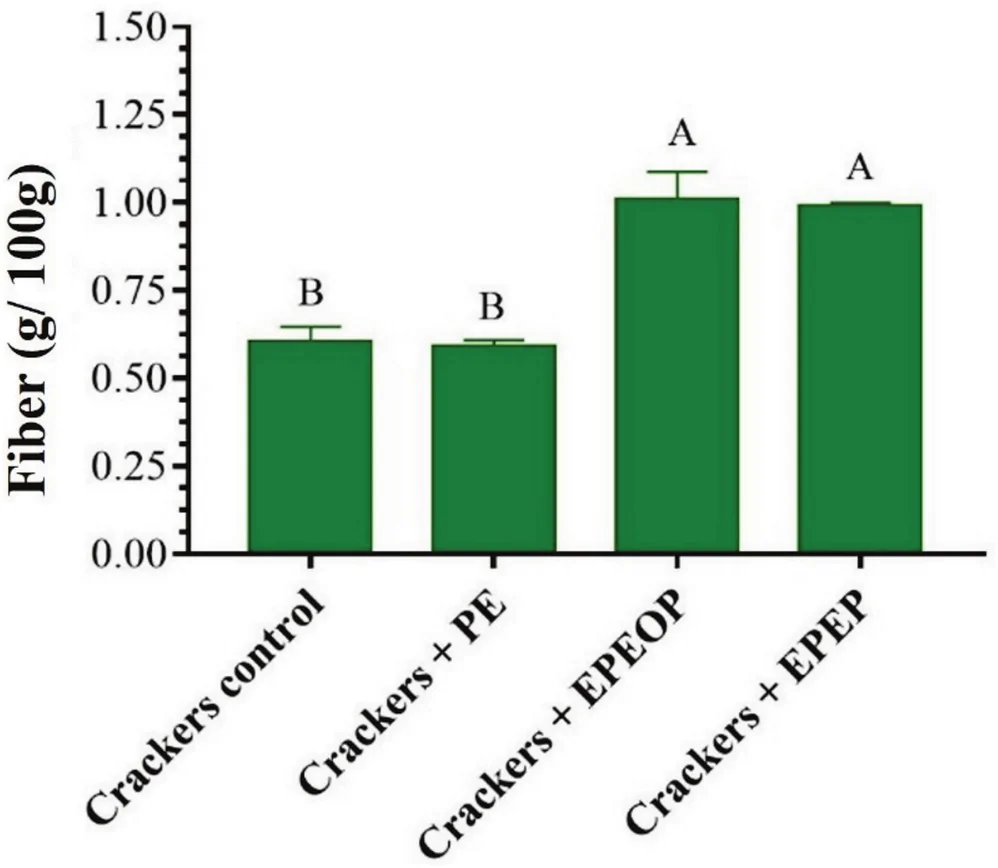
Fiber for crackers enriched with nonencapsulated PE and encapsulated extract (EPEOP and EPEP), PE, pomegranate extract; EPEOP, encapsulated pomegranate extract in orange peel; EPEP, encapsulated pomegranate extract in pectin. Capital letters (B and A) indicate differences between treatments according to the independent‐samples *t*‐test. Significant differences were determined according to Duncan's post hoc test (*p* < 0.05).

Figure 8 presents the total fiber content (g/100 g) in unfortified crackers (Crackers Control), crackers fortified with non‐encapsulated pomegranate extract (Crackers +PE), and crackers fortified with encapsulated pomegranate extract in orange peel (Crackers +EPEOP) and pectin (Crackers +EPEP). The results indicate highly significant differences in fiber content among the different cracker formulations (*p* < 0.001). Crackers fortified with encapsulated extracts, namely Crackers +EPEOP (1.01 ± 0.08 g/100 g) and Crackers +EPEP (0.99 ± 0.01 g/100 g), exhibited significantly higher fiber content compared to both the unfortified control crackers (0.61 ± 0.04 g/100 g) and crackers fortified with non‐encapsulated PE (0.59 ± 0.02 g/100 g). There were no significant differences in fiber content between Crackers Control and Crackers +PE. This clear distinction highlights the direct contribution of the encapsulating materials—orange peel and pectin—to the overall fiber content of the fortified crackers.

The increased fiber content in Crackers +EPEOP is directly attributable to the high dietary fiber content naturally present in orange peel. Orange peel is a rich source of both soluble and insoluble dietary fibers, including cellulose, hemicellulose, and pectin (Liu et al. 2025; Panić et al. 2021). Its incorporation as an encapsulating material not only provides a protective matrix for the phenolic compounds but also enhances the nutritional profile of the final product by boosting its fiber content. Similarly, pectin, used in Crackers +EPEP, is a well‐known soluble dietary fiber (Weber et al. 2025). Its addition as an encapsulating agent directly contributes to the increased fiber content in these crackers. This dual benefit of encapsulation—protection of bioactive compounds and nutritional enhancement—underscores the value of utilizing these natural materials.

### 3.9 Sensory Evaluation of Fortified Crackers

**FIGURE 9:**
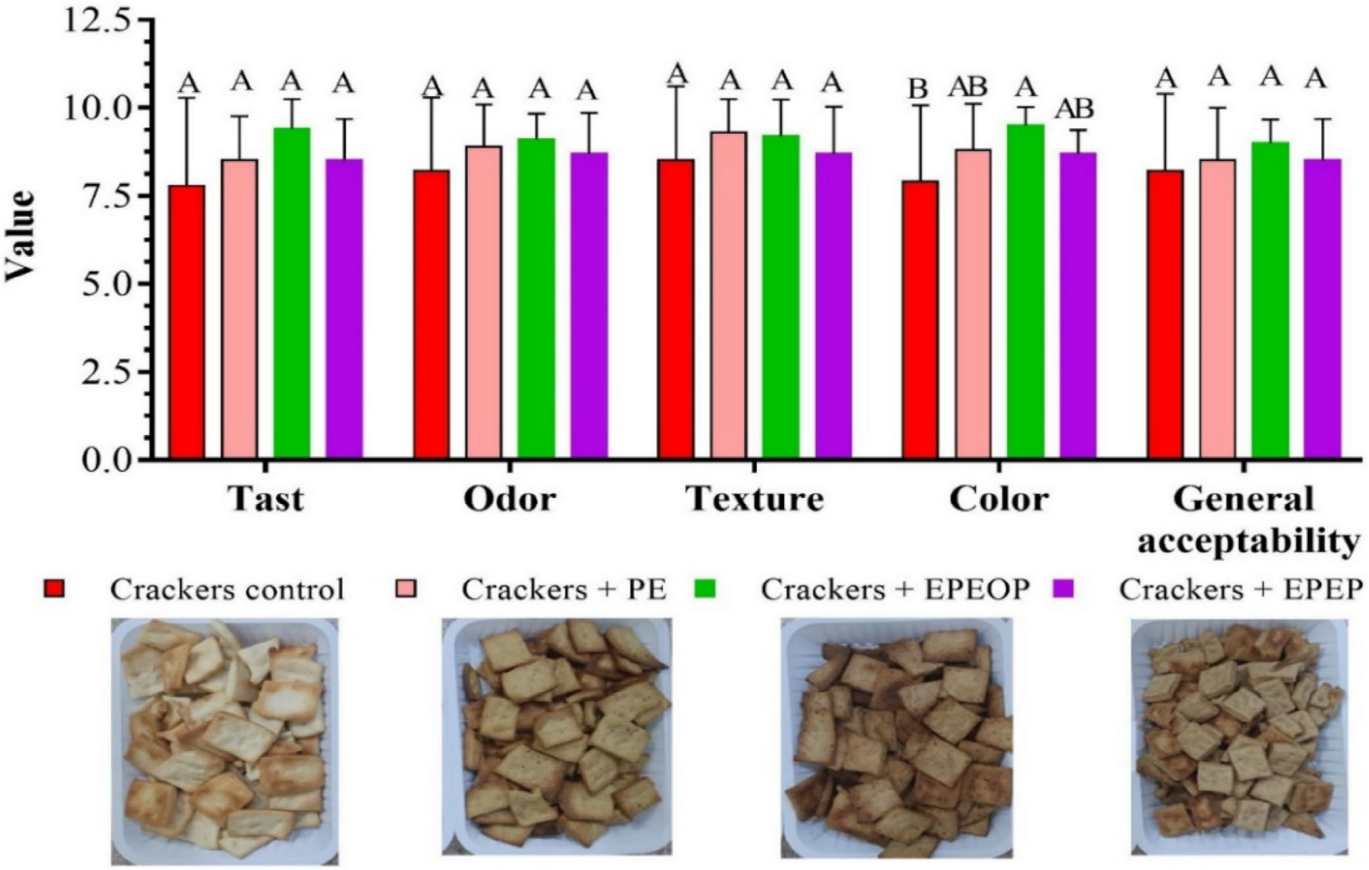
Sensory Evaluation for crackers enriched with nonencapsulated PE and encapsulated extract (EPEOP and EPEP), PE, pomegranate extract; EPEOP, encapsulated pomegranate extract in orange peel; EPEP, encapsulated pomegranate extract in pectin. Capital letters (B and A) indicate differences between the treatments according to the independent‐samples *t*‐test. Significant differences were determined according to Duncan's post hoc test (*p* < 0.05).

Figure 9 presents the sensory evaluation results of crackers fortified with non‐encapsulated pomegranate peel extract (PE) and encapsulated extracts (EPEOP and EPEP) compared with the control crackers. The evaluated attributes included taste, aroma, texture, color, and overall acceptability, using a scoring scale in which higher values indicate better sensory quality. The results showed no significant differences among samples in taste, aroma, or texture. In contrast, significant differences were observed in color. Crackers fortified with the orange‐peel encapsulated extract (EPEOP) obtained the highest scores among the tested samples, particularly for color (9.50 ± 0.53) and overall acceptability (9.00 ± 0.67). These findings agree with the instrumental color results discussed earlier in Section 3.7, where Crackers +EPEOP showed the greatest color stability.

Overall, the incorporation of pomegranate peel extract did not negatively affect the sensory properties of the crackers, regardless of whether the extract was encapsulated or not. In some cases, sensory quality was slightly improved, particularly in crackers containing EPEOP. Similar observations were reported by Kaderides et al. (2020), who indicated that phenolic extracts can be incorporated into bakery products without negatively affecting sensory quality.

Encapsulation may also contribute to improving sensory perception by moderating the release of polyphenolic compounds that are often associated with bitter or astringent flavors. By controlling their release, microencapsulation can reduce undesirable taste intensity and maintain a more balanced flavor profile (Ozkan et al. 2019). Previous studies on bakery products enriched with encapsulated polyphenols have likewise reported good sensory acceptance and improved product stability (Bińkowska et al. 2024; Kaewmaanee and Jennifer 2020). Similarly, consumer acceptance studies have shown that bakery products fortified with encapsulated phenolic compounds generally achieve favorable sensory scores while offering additional nutritional benefits (Abozed et al. 2024; Ciont et al. 2023). These findings support the results of the present study, where crackers fortified with encapsulated extracts—particularly EPEOP—showed high sensory scores and strong overall acceptability, suggesting good potential for functional bakery applications.

## 4 Conclusion

The present study evaluated the performance of an orange peel‐based encapsulation system developed using a patented two‐stage lyophilization technique for improving the stability of pomegranate peel phenolic compounds during storage and baking. Compared with the commercial pectin‐based encapsulation system, the orange peel‐based system showed superior retention of bioactive compounds and contributed to improved color stability, increased dietary fiber content, and favorable sensory acceptability in fortified crackers. However, the encapsulated extracts did not provide greater inhibition of primary lipid oxidation than the non‐encapsulated extract under accelerated storage conditions (60°C), suggesting that additional preservation strategies may be required for long‐term oxidative stability. A limitation of the present study is that encapsulation efficiency and the distribution of bound and free phenolic compounds were not determined, as the study focused on evaluating the functional performance of the encapsulated extracts during storage and baking rather than characterizing the encapsulation process itself. Other relevant technological characteristics of the encapsulated powders, including process yield, moisture content, water activity, solubility, and particle characteristics, were also not evaluated. In addition, the present study was not designed to distinguish the individual contributions of the wall material and the encapsulation strategy. Therefore, the superior performance of the orange peel‐based system should be interpreted as the combined effect of both factors. Future studies should address these limitations by characterizing encapsulation efficiency, bound and free phenolic fractions, and relevant physicochemical and technological properties, comparing wall materials using identical encapsulation procedures, and evaluating the encapsulated extracts under realistic storage conditions and in different food matrices.

## Author Contributions

**Marwa Ezz El‐Din Ibrahim:** conceptualization, methodology, software, data curation, investigation, funding acquisition, writing – original draft, validation, visualization, writing – review and editing, project administration, formal analysis, supervision, resources. **Randah M. Alqurashi:** methodology, validation, visualization, formal analysis, resources, funding acquisition, investigation, writing – review and editing.

## Funding

This work was supported by the Deanship of Scientific Research, Vice Presidency for Graduate Studies and Scientific Research, King Faisal University, Saudi Arabia (KFU263058).

## Disclosure

Patents: The concept underlying this research is protected by a U.S. patent (No. US11864574B1, United States).

## Ethics Statement

The sensory evaluation was approved by the Research Ethics Committee of King Faisal University (Approval No. KFU‐REC‐ETHICS4344).

## Conflicts of Interest

The authors declare no conflicts of interest.

## Supporting information


**Table S1:** Total phenolics content of nonencapsulated PE and encapsulated extract (EPEOP and EPEP) during storage at 60°C.
**Table S2:** Antioxidant activity of nonencapsulated PE and encapsulated extract (EPEOP and EPEP) during storage at 60°C.
**Table S3:** Total phenol content for crackers enriched with nonencapsulated PE and encapsulated extract (EPEOP and EPEP) during storage at 60°C.
**Table S4:** Antioxidant activity (%) for crackers enriched with nonencapsulated PE and encapsulated extract (EPEOP and EPEP) during storage at 60°C.

## Data Availability

The original contributions presented in the study are included in the article; further inquiries can be directed to the corresponding authors.

## References

[fsn372311-bib-0001] Abdel‐Aal, E.‐S. M. , and I. Rabalski . 2013. “Effect of Baking on Free and Bound Phenolic Acids in Wholegrain Bakery Products.” Journal of Cereal Science 57, no. 3: 312–318. 10.1016/j.jcs.2012.12.001.

[fsn372311-bib-0002] Abdel‐Aal, E.‐S. M. , and I. Rabalski . 2022. “Changes in Phenolic Acids and Antioxidant Properties During Baking of Bread and Muffin Made From Blends of Hairless Canary Seed, Wheat, and Corn.” Antioxidants 11, no. 6: 1059. 10.3390/antiox11061059. 35739956 PMC9220130

[fsn372311-bib-0003] Abozed, S. S. , Z. S. Ahmed , A. E. Abd El‐Kader , and R. K. Mohamed . 2024. “Characterization of Physicochemical, Antioxidant, and Sensory Quality of Cookies Enriched With Microencapsulated Ginger Extracts as a Functional Ingredient.” Egyptian Journal of Chemistry 67, no. 8: 525–535. 10.21608/ejchem.2024.239223.8714.

[fsn372311-bib-0004] Akonjuen, B. M. , and A. N. A. Aryee . 2023. “Novel Extraction and Encapsulation Strategies for Food Bioactive Lipids to Improve Stability and Control Delivery.” Food Chemistry Advances 2: 100278. 10.1016/j.focha.2023.100278.

[fsn372311-bib-0005] Alqurashi, R. , D. Commane , and I. Rowland . 2016. “Açai Fruit as a Source of Bioactive Phytochemicals.” Journal of Life Sciences 10: 391–404. 10.17265/1934-7391/2016.08.005.

[fsn372311-bib-0006] Andrade, M. A. , C. H. Barbosa , M. A. Shah , et al. 2022. “Citrus By‐Products: Valuable Source of Bioactive Compounds for Food Applications.” Antioxidants 12, no. 1: 38. 10.3390/antiox12010038. 36670900 PMC9855225

[fsn372311-bib-0086] AOAC . 2000. Official Methods of Analysis of AOAC International. 17th ed. AOAC International.

[fsn372311-bib-0007] Assadpour, E. , S.‐M. Jafari , and Y. Maghsoudlou . 2017. “Evaluation of Folic Acid Release From Spray Dried Powder Particles of Pectin‐Whey Protein Nano‐Capsules.” International Journal of Biological Macromolecules 95: 238–247. 10.1016/j.ijbiomac.2016.11.023. 27840216

[fsn372311-bib-0008] Bae, I. K. , H. M. Ham , M. H. Jeong , D. H. Kim , and H. J. Kim . 2015. “Simultaneous Determination of 15 Phenolic Compounds and Caffeine in Teas and Mate Using RP‐HPLC/UV Detection: Method Development and Optimization of Extraction Process.” Food Chemistry 172: 469–475. 10.1016/j.foodchem.2014.09.050. 25442580

[fsn372311-bib-0009] Ballesteros, L. F. , M. J. Ramirez , C. E. Orrego , J. A. Teixeira , and S. I. Mussatto . 2017. “Encapsulation of Antioxidant Phenolic Compounds Extracted From Spent Coffee Grounds by Freeze‐Drying and Spray‐Drying Using Different Coating Materials.” Food Chemistry 237: 623–631. 10.1016/j.foodchem.2017.05.142. 28764044

[fsn372311-bib-0010] Benchagra, L. , H. Berrougui , M. O. Islam , et al. 2021. “Antioxidant Effect of Moroccan Pomegranate ( *Punica granatum* L. Sefri Variety) Extracts Rich in Punicalagin Against the Oxidative Stress Process.” Food 10, no. 9: 2219. 10.3390/foods10092219. PMC8469689 34574329

[fsn372311-bib-0011] Bińkowska, W. , A. Szpicer , A. Stelmasiak , I. Wojtasik‐Kalinowska , and A. Półtorak . 2024. “Microencapsulation of Polyphenols and Their Application in Food Technology.” Applied Sciences 14, no. 24: 11954. 10.3390/app142411954.

[fsn372311-bib-0012] Çam, M. , and N. C. İçyer . 2015. “Phenolics of Pomegranate Peels: Extraction Optimization by Central Composite Design and Alpha Glucosidase Inhibition Potentials.” Journal of Food Science and Technology 52, no. 3: 1489–1497. 10.1007/s13197-013-1148-y. 25745217 PMC4348292

[fsn372311-bib-0013] Cano‐Lamadrid, M. , F. Hernández , P. Nowicka , A. A. Carbonell‐Barrachina , and A. Wojdyło . 2018. “Formulation and Storage Effects on Pomegranate Smoothie Phenolic Composition, Antioxidant Capacity and Color.” LWT 96: 322–328. 10.1016/j.lwt.2018.05.047.

[fsn372311-bib-0014] Cao, H. , O. Saroglu , A. Karadag , et al. 2021. “Available Technologies on Improving the Stability of Polyphenols in Food Processing.” Food Frontiers 2, no. 2: 109–139. 10.1002/fft2.65.

[fsn372311-bib-0016] Chezanoglou, E. , N. Kenanidou , C. Spyropoulos , D. Xenitopoulou , E. Zlati , and A. M. Goula . 2023. “Encapsulation of Pomegranate Peel Extract in Sucrose Matrix by Co‐Crystallization.” Sustainable Chemistry and Pharmacy 31: 100949. 10.1016/j.scp.2022.100949.

[fsn372311-bib-0017] Choi, M.‐Y. , C. Chai , J. H. Park , J. Lim , J. Lee , and S. W. Kwon . 2011. “Effects of Storage Period and Heat Treatment on Phenolic Compound Composition in Dried Citrus Peels (Chenpi) and Discrimination of Chenpi With Different Storage Periods Through Targeted Metabolomic Study Using HPLC‐DAD Analysis.” Journal of Pharmaceutical and Biomedical Analysis 54, no. 4: 638–645. 10.1016/j.jpba.2010.09.036. 21145683

[fsn372311-bib-0018] Choudhury, N. , M. Meghwal , and K. Das . 2021. “Microencapsulation: An Overview on Concepts, Methods, Properties and Applications in Foods.” Food Frontiers 2, no. 4: 426–442. 10.1002/fft2.94.

[fsn372311-bib-0019] Ciont, C. , G. Difonzo , A. Pasqualone , et al. 2023. “Phenolic Profile of Micro‐ and Nano‐Encapsulated Olive Leaf Extract in Biscuits During In Vitro Gastrointestinal Digestion.” Food Chemistry 428: 136778. 10.1016/j.foodchem.2023.136778. 37421669

[fsn372311-bib-0020] Ciurzyńska, A. , and A. Lenart . 2011. “Freeze‐Drying—Application in Food Processing and Biotechnology—A Review.” Polish Journal of Food and Nutrition Sciences 61, no. 3: 165–171. 10.2478/v10222-011-0017-5.

[fsn372311-bib-0021] Cortez‐Trejo, M. C. , A. Wall‐Medrano , M. Gaytán‐Martínez , and S. Mendoza . 2021. “Microencapsulation of Pomegranate Seed Oil Using a Succinylated Taro Starch: Characterization and Bioaccessibility Study.” Food Bioscience 41: 100929. 10.1016/j.fbio.2021.100929.

[fsn372311-bib-0022] Costa, N. H. B. , G. B. Amadio , Y. J. Souza‐Santos , R. A. Carvalho , and F. M. Vanin . 2025. “Incorporation of Pomegranate ( *Punica granatum* ) Extract Enhanced the Concentration of Bioactive Compounds and Shelf Life of Bread.” Food Science & Nutrition 13, no. 7: e70690. 10.1002/fsn3.70690. 40708778 PMC12288372

[fsn372311-bib-0023] Da Silva Júnior, M. E. , M. V. R. L. Araújo , A. C. S. Martins , et al. 2023. “Microencapsulation by Spray‐Drying and Freeze‐Drying of Extract of Phenolic Compounds Obtained From Ciriguela Peel.” Scientific Reports 13, no. 1: 15222. 10.1038/s41598-023-40390-4. 37709786 PMC10502068

[fsn372311-bib-0024] Das, A. K. , P. K. Nanda , N. R. Chowdhury , et al. 2021. “Application of Pomegranate By‐Products in Muscle Foods: Oxidative Indices, Colour Stability, Shelf Life and Health Benefits.” Molecules 26, no. 2: 467. 10.3390/molecules26020467. 33477314 PMC7830841

[fsn372311-bib-0025] De Freitas, V. A. P. , A. Fernandes , J. Oliveira , N. Teixeira , and N. Mateus . 2017. “A Review of the Current Knowledge of Red Wine Colour.” OENO One 51, no. 1: 1–15. 10.20870/oeno-one.2017.51.1.1604.

[fsn372311-bib-0026] Dueik, V. , and P. Bouchon . 2016. “Development of Polyphenol‐Enriched Vacuum and Atmospheric Fried Matrices: Evaluation of Quality Parameters and In Vitro Bioavailability of Polyphenols.” Food Research International 88: 166–172. 10.1016/j.foodres.2016.03.032. 28847397

[fsn372311-bib-0027] Elwers, S. , A. Zambrano , C. Rohsius , and R. Lieberei . 2009. “Differences Between the Content of Phenolic Compounds in Criollo, Forastero and Trinitario Cocoa Seed ( *Theobroma cacao* L.).” European Food Research and Technology 229, no. 6: 937–948. 10.1007/s00217-009-1132-y.

[fsn372311-bib-0028] Esfanjani, A. F. , S. M. Jafari , E. Assadpoor , and A. Mohammadi . 2015. “Nano‐Encapsulation of Saffron Extract Through Double‐Layered Multiple Emulsions of Pectin and Whey Protein Concentrate.” Journal of Food Engineering 165: 149–155. 10.1016/j.jfoodeng.2015.06.022.

[fsn372311-bib-0029] Ezhilarasi, P. N. , P. Karthik , N. Chhanwal , and C. Anandharamakrishnan . 2013. “Nanoencapsulation Techniques for Food Bioactive Components: A Review.” Food and Bioprocess Technology 6, no. 3: 628–647. 10.1007/s11947-012-0944-0.

[fsn372311-bib-0030] Fang, Z. , and B. Bhandari . 2010. “Encapsulation of Polyphenols – A Review.” Trends in Food Science & Technology 21, no. 10: 510–523. 10.1016/j.tifs.2010.08.003.

[fsn372311-bib-0031] Fathi, M. , Á. Martín , and D. J. McClements . 2014. “Nanoencapsulation of Food Ingredients Using Carbohydrate Based Delivery Systems.” Trends in Food Science & Technology 39, no. 1: 18–39. 10.1016/j.tifs.2014.06.007.

[fsn372311-bib-0032] Fawole, O. A. , and U. L. Opara . 2016. “Stability of Total Phenolic Concentration and Antioxidant Capacity of Extracts From Pomegranate Co‐Products Subjected to In Vitro Digestion.” BMC Complementary and Alternative Medicine 16, no. 1: 358. 10.1186/s12906-016-1343-2. 27618992 PMC5020488

[fsn372311-bib-0033] Ghasemi, S. , S. M. Jafari , E. Assadpour , and M. Khomeiri . 2017. “Production of Pectin‐Whey Protein Nano‐Complexes as Carriers of Orange Peel Oil.” Carbohydrate Polymers 177: 369–377. 10.1016/j.carbpol.2017.09.009. 28962781

[fsn372311-bib-0034] Grgić, J. , G. Šelo , M. Planinić , M. Tišma , and A. Bucić‐Kojić . 2020. “Role of the Encapsulation in Bioavailability of Phenolic Compounds.” Antioxidants 9, no. 10: 923. 10.3390/antiox9100923. 32993196 PMC7601682

[fsn372311-bib-0035] Gumienna, M. , A. Szwengiel , and B. Górna . 2016. “Bioactive Components of Pomegranate Fruit and Their Transformation by Fermentation Processes.” European Food Research and Technology 242, no. 5: 631–640. 10.1007/s00217-015-2582-z.

[fsn372311-bib-0036] Huang, Z. , S. C. Foo , and W. S. Choo . 2025. “A Review on the Extraction of Polyphenols From Pomegranate Peel for Punicalagin Purification: Techniques, Applications, and Future Prospects.” Sustainable Food Technology 3, no. 2: 396–413. 10.1039/D4FB00304G.

[fsn372311-bib-0037] Ibrahim, E. , and R. Alqurashi . 2024. “Encapsulated Polyphenolic Compounds.” (Patent No. US11864574B1). https://patents.google.com/patent/US11864574B1/en.

[fsn372311-bib-0038] Insang, S. , I. Kijpatanasilp , S. Jafari , and K. Assatarakul . 2022. “Ultrasound‐Assisted Extraction of Functional Compound From Mulberry ( *Morus alba* L.) Leaf Using Response Surface Methodology and Effect of Microencapsulation by Spray Drying on Quality of Optimized Extract.” Ultrasonics Sonochemistry 82: 105806. 10.1016/j.ultsonch.2021.105806. 34991963 PMC8799475

[fsn372311-bib-0039] Jakobek, L. 2015. “Interactions of Polyphenols With Carbohydrates, Lipids and Proteins.” Food Chemistry 175: 556–567. 10.1016/j.foodchem.2014.12.013. 25577120

[fsn372311-bib-0040] Kaderides, K. , and A. M. Goula . 2017. “Development and Characterization of a New Encapsulating Agent From Orange Juice By‐Products.” Food Research International 100: 612–622. 10.1016/j.foodres.2017.07.057. 28873728

[fsn372311-bib-0041] Kaderides, K. , A. M. Goula , and K. G. Adamopoulos . 2015. “A Process for Turning Pomegranate Peels Into a Valuable Food Ingredient Using Ultrasound‐Assisted Extraction and Encapsulation.” Innovative Food Science & Emerging Technologies 31: 204–215. 10.1016/j.ifset.2015.08.006.

[fsn372311-bib-0042] Kaderides, K. , A. Kyriakoudi , I. Mourtzinos , and A. M. Goula . 2021. “Potential of Pomegranate Peel Extract as a Natural Additive in Foods.” Trends in Food Science & Technology 115: 380–390. 10.1016/j.tifs.2021.06.050.

[fsn372311-bib-0043] Kaderides, K. , I. Mourtzinos , and A. M. Goula . 2020. “Stability of Pomegranate Peel Polyphenols Encapsulated in Orange Juice Industry By‐Product and Their Incorporation in Cookies.” Food Chemistry 310: 125849. 10.1016/j.foodchem.2019.125849. 31753686

[fsn372311-bib-0044] Kaewmaanee, T. , and A. Jennifer . 2020. “Storage Stability, Gastrointestinal Release and Sensory Properties of Cookies Incorporated With Protein‐Based *Moringa oleifera* Leaf Extract Microcapsule.” Chiang Mai University Journal of Natural Sciences 19, no. 1: 139–154. 10.12982/CMUJNS.2020.0009.

[fsn372311-bib-0045] Kandylis, P. , and E. Kokkinomagoulos . 2020. “Food Applications and Potential Health Benefits of Pomegranate and Its Derivatives.” Food 9, no. 2: 122. 10.3390/foods9020122. PMC7074153 31979390

[fsn372311-bib-0046] Khoddami, A. , M. A. Wilkes , and T. H. Roberts . 2013. “Techniques for Analysis of Plant Phenolic Compounds.” Molecules 18, no. 2: 2328–2375. 10.3390/molecules18022328. 23429347 PMC6270361

[fsn372311-bib-0047] Ledari, S. A. , J. M. Milani , S.‐A. Shahidi , and A. Golkar . 2024. “Comparative Analysis of Freeze Drying and Spray Drying Methods for Encapsulation of Chlorophyll With Maltodextrin and Whey Protein Isolate.” Food Chemistry: X 21: 101156. 10.1016/j.fochx.2024.101156. 38322765 PMC10844667

[fsn372311-bib-0048] Liu, Z. , M. Zhang , and Z. Luo . 2025. “Effect of Wall Materials on Physicochemical Properties and Bitterness Masking of Freeze‐Dried Navel Orange Peel Powder.” Food Bioscience 66: 106226. 10.1016/j.fbio.2025.106226.

[fsn372311-bib-0049] Luca, A. , B. Cilek , V. Hasirci , S. Sahin , and G. Sumnu . 2014. “Storage and Baking Stability of Encapsulated Sour Cherry Phenolic Compounds Prepared From Micro‐ and Nano‐Suspensions.” Food and Bioprocess Technology 7, no. 1: 204–211. 10.1007/s11947-013-1048-1.

[fsn372311-bib-0050] Maier, T. , M. Fromm , S. Andreas , K. Dietmar , and R. Carle . 2009. “Process and Storage Stability of Anthocyanins and Non‐Anthocyanin Phenolics in Pectin and Gelatin Gels Enriched With Grape Pomace Extracts.” Uropean Food Research and Technology 229: 949–960. 10.1007/s00217-009-1134-9.

[fsn372311-bib-0051] Maire, M. , B. Rega , M.‐E. Cuvelier , P. Soto , and P. Giampaoli . 2013. “Lipid Oxidation in Baked Products: Impact of Formula and Process on the Generation of Volatile Compounds.” Food Chemistry 141, no. 4: 3510–3518. 10.1016/j.foodchem.2013.06.039. 23993514

[fsn372311-bib-0052] Man, G. , L. Xu , Y. Wang , X. Liao , and Z. Xu . 2022. “Profiling Phenolic Composition in Pomegranate Peel From Nine Selected Cultivars Using UHPLC‐QTOF‐MS and UPLC‐QQQ‐MS.” Frontiers in Nutrition 8: 807447. 10.3389/fnut.2021.807447. 35141267 PMC8819070

[fsn372311-bib-0053] Manley, D. 2011. “Product Development in the Biscuit Industry.” In Manley's Technology of Biscuits, Crackers and Cookies, 4th ed., 69–92. Elsevier (Original work published Woodhead Publishing Series in Food Science, Technology and Nutrition). 10.1533/9780857093646.1.69.

[fsn372311-bib-0055] McCain‐Keefer, H. R. , S. Meals , and M. Drake . 2020. “The Sensory Properties and Consumer Acceptance of Cold Brew Coffee.” Journal of Sensory Studies 35, no. 6: e12604. 10.1111/joss.12604.

[fsn372311-bib-0056] Moreno, T. , M. J. Cocero , and S. Rodríguez‐Rojo . 2018. “Storage Stability and Simulated Gastrointestinal Release of Spray Dried Grape Marc Phenolics.” Food and Bioproducts Processing 112: 96–107. 10.1016/j.fbp.2018.08.011.

[fsn372311-bib-0057] Moser, P. , V. R. N. Telis , N. De Andrade Neves , E. García‐Romero , S. Gómez‐Alonso , and I. Hermosín‐Gutiérrez . 2017. “Storage Stability of Phenolic Compounds in Powdered BRS Violeta Grape Juice Microencapsulated With Protein and Maltodextrin Blends.” Food Chemistry 214: 308–318. 10.1016/j.foodchem.2016.07.081. 27507480

[fsn372311-bib-0058] Mousumi, A. , A. K. M. A. Nowsad , M. I. Hossain , and A. Shahriar . 2025. “Comparative Study on the Use of Antioxidants Rich Vegetables and First Evidence of Carotenoids and Phenolic Compounds From Fresh Carrot as Natural Antioxidants to Stabilize Lipid in Highly Fatty Hilsa Shad ( *Tenualosa ilisha* ).” Applied Food Research 5, no. 1: 100979. 10.1016/j.afres.2025.100979.

[fsn372311-bib-0059] Ozkan, G. , P. Franco , I. De Marco , J. Xiao , and E. Capanoglu . 2019. “A Review of Microencapsulation Methods for Food Antioxidants: Principles, Advantages, Drawbacks and Applications.” Food Chemistry 272: 494–506. 10.1016/j.foodchem.2018.07.205. 30309574

[fsn372311-bib-0060] Panić, M. , M. Andlar , M. Tišma , et al. 2021. “Natural Deep Eutectic Solvent as a Unique Solvent for Valorisation of Orange Peel Waste by the Integrated Biorefinery Approach.” Waste Management 120: 340–350. 10.1016/j.wasman.2020.11.052. 33340816

[fsn372311-bib-0061] Rahul, P. B. , R. K. Tiwari , K. K. Dash , and M. Sharma . 2025. “Recent Advances in Encapsulation of Pomegranate Peel Extract and Combination of Wall Materials: A Review of Encapsulation Technologies, Characterization and Applications in the Food Industry.” Sustainable Food Technology 3, no. 1: 123–144. 10.1039/d4fb00196f.

[fsn372311-bib-0062] Ratti, C. 2001. “Hot Air and Freeze‐Drying of High‐Value Foods: A Review.” Journal of Food Engineering 49, no. 4: 311–319. 10.1016/S0260-8774(00)00228-4.

[fsn372311-bib-0063] Robert, P. , V. Torres , P. García , C. Vergara , and C. Sáenz . 2015. “The Encapsulation of Purple Cactus Pear ( *Opuntia ficus‐indica* ) Pulp by Using Polysaccharide‐Proteins as Encapsulating Agents.” LWT ‐ Food Science and Technology 60, no. 2: 1039–1045. 10.1016/j.lwt.2014.10.038.

[fsn372311-bib-0064] Rocha‐Parra, D. F. , M. C. Lanari , M. C. Zamora , and J. Chirife . 2016. “Influence of Storage Conditions on Phenolic Compounds Stability, Antioxidant Capacity and Colour of Freeze‐Dried Encapsulated Red Wine.” LWT 70: 162–170. 10.1016/j.lwt.2016.02.038.

[fsn372311-bib-0065] Różańska, M. B. , A. Siger , A. Szwengiel , K. Dziedzic , and S. Mildner‐Szkudlarz . 2021. “Maillard Reaction Products in Gluten‐Free Bread Made From Raw and Roasted Buckwheat Flour.” Molecules 26, no. 5: 1361. 10.3390/molecules26051361. 33806318 PMC7961691

[fsn372311-bib-0066] Saenz, C. , S. Tapia , J. Chavez , and P. Robert . 2009. “Microencapsulation by Spray Drying of Bioactive Compounds From Cactus Pear ( *Opuntia ficus‐indica* ).” Food Chemistry 114, no. 2: 616–622. 10.1016/j.foodchem.2008.09.095.

[fsn372311-bib-0067] Sanhueza, L. , P. García , B. Giménez , J. M. Benito , M. Matos , and G. Gutiérrez . 2022. “Encapsulation of Pomegranate Peel Extract ( *Punica granatum* L.) by Double Emulsions: Effect of the Encapsulation Method and Oil Phase.” Food 11, no. 3: 310. 10.3390/foods11030310. PMC8833941 35159459

[fsn372311-bib-0068] Sapers, G. M. , and F. W. Douglas . 1987. “Measurement of Enzymatic Browning at Cut Surfaces and in Juice of Raw Apple and Pear Fruits.” Journal of Food Science 52, no. 5: 1258–1285. 10.1111/j.1365-2621.1987.tb14057.x.

[fsn372311-bib-0069] Šaponjac, V. T. , G. Ćetković , S. Đilas , et al. 2017. “Encapsulation of Sour Cherry Pomace Extract by Freeze Drying: Characterization and Storage Stability.” Acta Chimica Slovenica 64, no. 2: 283–289. 10.17344/acsi.2016.2789. 28621401

[fsn372311-bib-0070] Schutte, M. , S. Hayward , and M. Manley . 2024. “Nonenzymatic Browning and Antioxidant Properties of Thermally Treated Cereal Grains and End Products.” Journal of Food Biochemistry 2024: 1–20. 10.1155/2024/3865849.

[fsn372311-bib-0071] Selcuk, N. , and M. Erkan . 2015. “Changes in Phenolic Compounds and Antioxidant Activity of Sour–Sweet Pomegranates cv. ‘Hicaznar’ During Long‐Term Storage Under Modified Atmosphere Packaging.” Postharvest Biology and Technology 109: 30–39. 10.1016/j.postharvbio.2015.05.018.

[fsn372311-bib-0072] Singh, J. , H. P. Kaur , A. Verma , et al. 2023. “Pomegranate Peel Phytochemistry, Pharmacological Properties, Methods of Extraction, and Its Application: A Comprehensive Review.” ACS Omega 8, no. 39: 35452–35469. 10.1021/acsomega.3c02586. 37810640 PMC10551920

[fsn372311-bib-0073] Stabrauskiene, J. , L. Pudziuvelyte , and J. Bernatoniene . 2024. “Optimizing Encapsulation: Comparative Analysis of Spray‐Drying and Freeze‐Drying for Sustainable Recovery of Bioactive Compounds From *Citrus* × *paradisi* L. Peels.” Pharmaceuticals 17, no. 5: 596. 10.3390/ph17050596. 38794165 PMC11123762

[fsn372311-bib-0074] Tang, Y. , Y. Huang , M. Li , et al. 2024. “Balancing Maillard Reaction Products Formation and Antioxidant Activities for Improved Sensory Quality and Health Benefit Properties of Pan Baked Buns.” Food Research International 195: 114984. 10.1016/j.foodres.2024.114984. 39277245

[fsn372311-bib-0075] Toledo‐Merma, P. R. , M. H. Cornejo‐Figueroa , A. D. R. Crisosto‐Fuster , et al. 2022. “Phenolic Compounds Recovery From Pomegranate ( *Punica granatum* L.) By‐Products of Pressurized Liquid Extraction.” Food 11, no. 8: 1070. 10.3390/foods11081070. PMC9024887 35454656

[fsn372311-bib-0076] Tolve, R. , F. Bianchi , E. Lomuscio , L. Sportiello , and B. Simonato . 2022. “Current Advantages in the Application of Microencapsulation in Functional Bread Development.” Food 12, no. 1: 96. 10.3390/foods12010096. PMC9818201 36613312

[fsn372311-bib-0077] Tolve, R. , F. Galgano , M. C. Caruso , et al. 2016. “Encapsulation of Health‐Promoting Ingredients: Applications in Foodstuffs.” International Journal of Food Sciences and Nutrition 67, no. 8: 888–918. 10.1080/09637486.2016.1205552. 27387656

[fsn372311-bib-0078] Tontul, I. , and A. Topuz . 2017. “Effects of Different Drying Methods on the Physicochemical Properties of Pomegranate Leather (Pestil).” LWT 80: 294–303. 10.1016/j.lwt.2017.02.035.

[fsn372311-bib-0079] Tsali, A. , and A. M. Goula . 2018. “Valorization of Grape Pomace: Encapsulation and Storage Stability of Its Phenolic Extract.” Powder Technology 340: 194–207. 10.1016/j.powtec.2018.09.011.

[fsn372311-bib-0080] Tumbas Šaponjac, V. , G. Ćetković , J. Čanadanović‐Brunet , et al. 2016. “Sour Cherry Pomace Extract Encapsulated in Whey and Soy Proteins: Incorporation in Cookies.” Food Chemistry 207: 27–33. 10.1016/j.foodchem.2016.03.082. 27080876

[fsn372311-bib-0081] Weber, A. M. , N. Pascale , F. Gu , E. P. Ryan , and F. Respondek . 2025. “Nutrition and Health Effects of Pectin: A Systematic Scoping Review of Human Intervention Studies.” Nutrition Research Reviews 38, no. 1: 306–323. 10.1017/S0954422424000180. 39324277

[fsn372311-bib-0082] Xu, L. , J.‐R. Cheng , X.‐M. Liu , and M.‐J. Zhu . 2019. “Effect of Microencapsulated Process on Stability of Mulberry Polyphenol and Oxidation Property of Dried Minced Pork Slices During Heat Processing and Storage.” LWT 100: 62–68. 10.1016/j.lwt.2018.10.025.

[fsn372311-bib-0083] Yan, L. , X. Zhou , L. Shi , D. Shalimu , C. Ma , and Y. Liu . 2017. “Phenolic Profiles and Antioxidant Activities of Six Chinese Pomegranate ( *Punica granatum* L.) Cultivars.” International Journal of Food Properties 20, no. sup1: S94–S107. 10.1080/10942912.2017.1289960.

[fsn372311-bib-0084] Yinbin, L. , L. Wu , M. Weng , B. Tang , P. Lai , and J. Chen . 2018. “Effect of Different Encapsulating Agent Combinations on Physicochemical Properties and Stability of Microcapsules Loaded With Phenolics of Plum ( *Prunus salicina* Lindl.).” Powder Technology 340: 459–464. 10.1016/j.powtec.2018.09.049.

[fsn372311-bib-0085] Žilić, S. , T. Kocadağlı , J. Vančetović , and V. Gökmen . 2016. “Effects of Baking Conditions and Dough Formulations on Phenolic Compound Stability, Antioxidant Capacity and Color of Cookies Made From Anthocyanin‐Rich Corn Flour.” LWT 65: 597–603. 10.1016/j.lwt.2015.08.057.

