# Supplementary material for: Encapsulation of Pomegranate Peel Phenolic Extract Using Orange Peel and Pectin: Stability Evaluation in Baked Products
Source: Food Sci Nutr. 2026 Sep 2;14(9):e72311. doi: 10.1002/fsn3.72311 (PMC13538958; doi:10.1002/fsn3.72311)
Supplement: Supplementary file 1 [file FSN3-14-e72311-s001.docx]

**Supplementary Table 1: Total phenolics content of nonencapsulated PE and encapsulated extract (EPEOP and EPEP) during storage at 60°C.**

| Total phenol content (mg/g sample) | | | | | |
| --- | --- | --- | --- | --- | --- |
| Storage day | | pomegranate extract (PE) | encapsulated pomegranate extract  in orange peel (EPEOP) | encapsulated pomegranate  extract in pectin  EPEP | P value |
| 0 | | 12.19±0.47^ijA^ | 13.24±1.55^kA^ | 13.77±1.86^efA^ | 0.435 |
| 3 | | 11.62±0.08^jC^ | 13.73±0.75^jkB^ | 15.28±0.79^efA^ | 0.001 |
| 6 | | 12.30±0.04^iC^ | 14.74±0.64^iA^ | 13.58±0.46^fB^ | 0.002 |
| 9 | | 12.49±0.31^iB^ | 14.40±0.57^ijAB^ | 15.39±1.60^eA^ | 0.031 |
| 12 | | 13.92±1.06^hA^ | 14.81±0.62^iA^ | 14.58±0.90^efA^ | 0.479 |
| 15 | | 16.58±0.03^fC^ | 21.68±0.27^efgA^ | 20.10±0.01^dB^ | <0.001 |
| 18 | | 19.43±0.03^bB^ | 26.26±0.72^bA^ | 25.86±2.62^aA^ | 0.003 |
| 21 | | 15.69±0.09^gC^ | 20.31±0.09^hB^ | 23.82±0.15^bA^ | <0.001 |
| 24 | | 18.03±0.13^deC^ | 20.74±0.13^ghB^ | 21.73±0.01^cA^ | <0.001 |
| 27 | | 18.34±0.14^deC^ | 21.19±0.10^fghB^ | 22.21±0.05^bcA^ | <0.001 |
| 30 | | 18.60±0.52^cdC^ | 27.88±0.11^aA^ | 23.12±0.03^bcB^ | <0.001 |
| 33 | | 18.97±0.10^bcC^ | 22.31±0.15^deB^ | 23.30±0.01^bcA^ | <0.001 |
| 36 | | 17.89±0.37^eC^ | 21.90±0.51^efB^ | 23.22±0.03^bcA^ | <0.001 |
| 39 | | 17.76±0.10^eB^ | 23.37±0.03^cA^ | 23.76±0.85^bA^ | <0.001 |
| 42 | | 20.98±0.09^aB^ | 21.03±0.10^fghB^ | 24.06±0.01^bA^ | <0.001 |
| 45 | | 17.13±0.04^fC^ | 23.16±0.06^cdA^ | 22.45±0.04^bcB^ | <0.001 |
| p value | Treatment | <0.001 | <0.001 | <0.001 |  |
|  | Linear | <0.001 | <0.001 | <0.001 |  |
|  | Quadratic | <0.001 | <0.001 | <0.001 |  |

Means± SD with same letters indicate non-significant differences, means with difference letters indicate statistically significant differences (p<0.05) according to Duncan's post hoc test. Lowercase letters (a & b) indicate differences between sample days in the same column. Capital letters (B & A) indicate differences between the treatment in the same row. PE: pomegranate extract, EPEOP: encapsulated pomegranate extract in orange peel, EPEP: encapsulated pomegranate extract in pectin

**Supplementary Table 2: Antioxidant activity of nonencapsulated PE and encapsulated extract (EPEOP and EPEP) during storage at 60°C**

| **Antioxidant activity (%)** | | | | | |
| --- | --- | --- | --- | --- | --- |
| Storage day | | pomegranate extract (PE) | encapsulated pomegranate extract  in orange peel (EPEOP) | encapsulated pomegranate  extract in pectin  EPEP | P value |
| 0 | | 77.80±0.37^bA^ | 81.53±1.04^eA^ | 68.03±3.10^gB^ | <0.001 |
| 3 | | 64.80±0.11^dC^ | 78.30±0.78^fA^ | 69.47±0.94^gB^ | <0.001 |
| 6 | | 37.94±0.28^kC^ | 72.45±0.39^hA^ | 57.52±0.92^iB^ | <0.001 |
| 9 | | 47.08±2.81^hC^ | 74.81±1.97^gA^ | 62.38±0.57^hB^ | <0.001 |
| 12 | | 78.17±1.48^bA^ | 69.34±0.66^iB^ | 51.24±1.56^jC^ | <0.001 |
| 15 | | 92.35±0.49^aC^ | 95.90±0.67^abB^ | 99.07±0.19^aA^ | <0.001 |
| 18 | | 61.38±0.19^eC^ | 68.22±1.63^iB^ | 88.74±2.08^eA^ | <0.001 |
| 21 | | 73.76±0.11^cB^ | 96.83±0.56^aA^ | 97.47±0.03^abA^ | <0.001 |
| 24 | | 74.88±0.28^cB^ | 95.40±0.47^abA^ | 95.52±0.49^bcA^ | <0.001 |
| 27 | | 64.99±0.47^dB^ | 96.39±1.09^abA^ | 85.14±1.69^fA^ | <0.001 |
| 30 | | 42.60±0.28^iC^ | 73.45±2.86^ghB^ | 92.97±3.61^cdA^ | <0.001 |
| 33 | | 40.61±1.03^jC^ | 91.79±0.19^cB^ | 95.27±0.28^bcA^ | <0.001 |
| 36 | | 49.38±0.11^gC^ | 92.16±1.31^cA^ | 62.69±1.87^hB^ | <0.001 |
| 39 | | 51.37±0.47^fC^ | 87.75±0.75^dB^ | 91.11±0.57^deA^ | <0.001 |
| 42 | | 22.82±1.09^lC^ | 97.14±0.28^aA^ | 92.23±1.06^dB^ | <0.001 |
| 45 | | 65.86±0.93^dB^ | 94.47±0.71^bA^ | 93.10±0.49^cdA^ | <0.001 |
| P value | Treatment | <0.001 | <0.001 | <0.001 |  |
|  | Linear | <0.001 | <0.001 | <0.001 |  |
|  | Quadratic | <0.001 | 0.488 | <0.001 |  |

Means± SD with same letters indicate non-significant differences, means with difference letters indicate statistically significant differences (p<0.05) according to Duncan's post hoc test. Lowercase letters (a & b) indicate differences between sample days in the same column. Capital letters (B & A) indicate differences between the treatment in the same row. PE: pomegranate extract, EPEOP: encapsulated pomegranate extract in orange peel, EPEP: encapsulated pomegranate extract in pectin.

**Supplementary Table 3: Total phenol content for crackers enriched with nonencapsulated PE and encapsulated extract (EPEOP and EPEP) during storage at 60°C**

| Total phenol content (mg/g sample) | | | | | | |
| --- | --- | --- | --- | --- | --- | --- |
| Items | | Crackers Control | Crackers +PE | Crackers + EPEOP | Crackers + EPEP | P value |
| 0 | | 0.21±0.07^cD^ | 1.45±0.08^dB^ | 1.95±0.03^deA^ | 0.63±0.04^cC^ | <0.001 |
| 3 | | 0.17±0.01^cC^ | 0.70±0.39^efB^ | 2.00±0.14^deA^ | 0.03±0.03^cC^ | <0.001 |
| 6 | | 0.39±0.36^cB^ | 1.11±0.15^defA^ | 1.17±0.09^efA^ | 0.15±0.03^cB^ | <0.001 |
| 9 | | 0.30±0.02^cC^ | 1.26±0.17^deA^ | 0.93±0.03^fB^ | 0.17±0.03^cC^ | <0.001 |
| 12 | | 0.37±0.12^cB^ | 0.51±0.43^fB^ | 1.58±0.02^efA^ | 0.87±0.35^cB^ | 0.004 |
| 15 | | 1.03±0.02^abB^ | 2.95±0.04^cA^ | 2.87±0.15^cdA^ | 4.45±1.63^abA^ | 0.007 |
| 18 | | 0.86±0.04^bC^ | 3.62±0.11^bB^ | 3.45±0.25^bcB^ | 4.88±0.99^abA^ | <0.001 |
| 21 | | 1.15±0.14^aB^ | 4.46±0.87^aA^ | 5.47±1.54^aA^ | 5.43±1.34^aA^ | 0.004 |
| 24 | | 1.16±0.13^aB^ | 4.07±0.35^abA^ | 4.18±0.27^bA^ | 3.74±1.35^bA^ | 0.002 |
| P value | Treatment | <0.001 | <0.001 | <0.001 | <0.001 |  |
|  | Linear | <0.001 | <0.001 | <0.001 | <0.001 |  |
|  | Quadratic | 0.089 | <0.001 | <0.001 | 0.001 |  |

Means± SD with same letters indicate non-significant differences, means with difference letters indicate statistically significant differences (p<0.05) according to Duncan's post hoc test. Lowercase letters (a & b) indicate differences between sample days in the same column. Capital letters (B & A) indicate differences between the treatment in the same row. PE: pomegranate extract, EPEOP: encapsulated pomegranate extract in orange peel, EPEP: encapsulated pomegranate extract in pectin

**Supplementary Table 4: Antioxidant activity (%) for crackers enriched with nonencapsulated PE and encapsulated extract (EPEOP and EPEP) during storage at 60°C**

| **Antioxidant activity (%)** | | | | | | |
| --- | --- | --- | --- | --- | --- | --- |
| Items | | Crackers Control | Crackers +PE | Crackers + EPEOP | Crackers + EPEP | P value |
| 0 | | 4.04±1.91^cdC^ | 51.80±1.81^aA^ | 21.27±2.05^eB^ | 6.53±1.71^cC^ | <0.001 |
| 3 | | 36.75±0.37^aB^ | 56.90±0.19^aA^ | 31.03±9.36^dBC^ | 24.69±3.45^bC^ | <0.001 |
| 6 | | 18.66±2.24^bC^ | 58.58±7.76^aA^ | 34.20±8.12^cdB^ | 2.67±2.06^cD^ | <0.001 |
| 9 | | 7.71±5.32^cC^ | 52.67±3.11^aA^ | 28.86±0.28^deB^ | 4.79±1.24^cC^ | <0.001 |
| 12 | | 32.77±3.83^aB^ | 51.68±1.53^aA^ | 32.65±0.99^cdB^ | 5.91±1.84^cC^ | <0.001 |
| 15 | | 0.62±0.60^dB^ | 55.41±2.12^aA^ | 46.08±1.49^bA^ | 42.10±19.49^aA^ | 0.001 |
| 18 | | 3.86±3.03^cdC^ | 39.74±4.13^bA^ | 40.86±4.04^bcA^ | 29.17±0.66^bB^ | <0.001 |
| 21 | | 1.37±0.39^dC^ | 17.23±4.58^cB^ | 41.73±6.73^bcA^ | 7.52±0.94^cC^ | <0.001 |
| 24 | | 3.23±2.83^cdD^ | 24.38±9.10^cC^ | 65.30±0.19^aA^ | 46.83±0.32^aB^ | <0.001 |
| P value | Treatment | <0.001 | <0.001 | <0.001 | <0.001 |  |
|  | Linear | <0.001 | <0.001 | <0.001 | <0.001 |  |
|  | Quadratic | <0.001 | <0.001 | 0.015 | 0.015 |  |

Means± SD with same letters indicate non-significant differences, means with difference letters indicate statistically significant differences (p<0.05) according to Duncan's post hoc test. Lowercase letters (a & b) indicate differences between sample days in the same column. Capital letters (B & A) indicate differences between the treatment in the same row. PE: pomegranate extract, EPEOP: encapsulated pomegranate extract in orange peel, EPEP: encapsulated pomegranate extract in pectin
